# Association between microRNA Polymorphisms and Cancer Risk Based on the Findings of 66 Case-Control Studies

**DOI:** 10.1371/journal.pone.0079584

**Published:** 2013-11-20

**Authors:** Xiao Pin Ma, Ting Zhang, Bo Peng, Long Yu, De Ke Jiang

**Affiliations:** 1 State Key Laboratory of Genetic Engineering, Institute of Genetics, School of Life Sciences, Fudan University, Shanghai, China; 2 Institute of Biomedical Science, Fudan University, Shanghai, China; 3 Fudan-VARI Center for Genetic Epidemiology, Fudan University, Shanghai, China; 4 Central Laboratory, The Affiliated Jiangyin Hospital of Southeast University Medical College, Jiangyin, Jiangsu, China; University of Aberdeen, United Kingdom

## Abstract

MicroRNAs (miRNAs) are small non-coding RNA molecules, which participate in diverse biological processes and may regulate tumor suppressor genes or oncogenes. Single nucleotide polymorphisms (SNPs) in miRNA may contribute to diverse functional consequences, including cancer development, by altering miRNA expression. Numerous studies have shown the association between miRNA SNPs and cancer risk; however, the results are generally debatable and inconclusive, mainly due to limited statistical power. To assess the relationship between the five most common SNPs (miR-146a rs2910164, miR-196a2 rs11614913, miR-499 rs3746444, miR-149 rs2292832, and miR-27a rs895919) and the risk cancer development, we performed a meta-analysis of 66 published case-control studies. Crude odds ratios at 95% confidence intervals were used to investigate the strength of the association. No association was observed between rs2910164 and cancer risk in the overall group. However, in stratified analysis, we found that either the rs2910164 C allele or the CC genotype was protective against bladder cancer, prostate cancer, cervical cancer, and colorectal cancer, whereas it was a risk factor for papillary thyroid carcinoma and squamous cell carcinoma of the head and neck (SCCHN). Further, rs11614913 was found to be significantly associated with decreased cancer risk, in particular, for bladder cancer, gastric cancer, and SCCHN. For miR-499, a significant association was found between the rs3746444 polymorphism and cancer risk in pooled analysis. In subgroup analysis, similar results were mainly observed for breast cancer. Finally, no association was found between rs2292832 and rs895919 polymorphisms and cancer risk in the overall group and in stratified analysis. In summary, miR-196a2 rs11614913, miR-146a rs2910164, and miR-499 rs3746444 are risk factors for cancer development, whereas mir-149 rs2292832 and miR-27a rs895919 are not associated with cancer risk.

## Introduction

Cancer is an outcome of unregulated expression of genes involved in development, cell growth, and differentiation. Many studies have shown that cancer is not only related to environmental factors, but also to individuals’ genetic susceptibility (predisposition). Recently, a new mechanism of microRNA (miRNA)-mediated transcriptional regulation was elucidated [1]. MiRNAs are a class of single-stranded short (21∼25 nt) RNAs, which are evolutionarily well conserved but are non–protein-coding. These RNAs regulate a broad range of biologic and pathologic process, including apoptosis, proliferation, differentiation, angiogenesis, and immune response, which are known to play critical roles in carcinogenesis [1]–[3]. MiRNAs bind to the 3′-untranslated region of the target mRNAs, leading to their degradation or translational suppression, thereby regulating the expression of target genes at the post-transcriptional level [2]. Estimates suggest that a single miRNA can target hundreds of mRNAs, and approximately 50% miRNA genes are located in cancer-related chromosomal regions [4]–[7]. Studies have shown that mature miRNAs regulate the expression of roughly 10–30% of all human genes [8]. Moreover, recent studies have suggested that miRNAs may participate in the carcinogenesis, progression (proliferation, migration, and invasion), and prognosis of multiple human malignancies by regulating the expression of tumor suppressor genes or proto-oncogenes [9]–[12].

Single nucleotide polymorphisms (SNPs) are the most common type of variation in the human genome, affecting sequence coding and splicing, which can influence the population diversity, disease susceptibility, and individual response to medicine [13]. SNPs can alter miRNA expression and/or maturation to affect function in three ways: through the transcription of the primary transcript, through pri-miRNA and pre-miRNA processing, and by affecting miRNA–mRNA interactions [14].

Many epidemiological studies have demonstrated the association of SNPs in miRNAs with the development and progression of cancer [14], [15]. MiR-146a rs2910164, miR-196a2 rs11614913, miR-499 rs3746444, miR-149 rs2292832, and miR-27a rs895919 are well-established miRNA polymorphisms [16]–[28] that have been reported to be associated with cancer risk [14]. However, conclusions of these studies remain inconsistent due to heterogeneity of the cancer subtype, limited sample size, and differences in the ethnicity of patients. To better assess the association of miR-146a rs2910164, miR-196a2 rs11614913, miR-499 rs3746444, miR-149 rs2292832, and miR-27a rs895919 in the miRNA genes with cancer risk, we conducted a meta-analysis of all eligible published case-control studies and evaluated the effect of the five SNPs on overall cancer risk. The effects of tumor type, ethnicity, source of controls, and sample size were also evaluated.

## Materials and Methods

### Publication Search

To identify all potentially eligible studies on miRNA polymorphisms and cancer risk, we carried out a systematic search on PubMed, Web of Science, Science Direct, and Embase, covering all papers published up to June 30, 2013, by using the search terms: “microRNA 146a/196a2/499/149/27a”, “mir-146a/196a2/499/27a”, “polymorphism”, and “cancer”. References of the retrieved articles and review articles were also screened. Eligible studies had to meet all of the following criteria: (a) full-text study, (b) evaluation of the association between miRNA polymorphisms and cancer risk, (c) unrelated case-control design, and (d) sufficient data for estimating the odds ratio (OR) with 95% confidence interval (CI) and a *P*-value. Studies containing two or more case-control groups were considered as two or more independent studies.

### Data Extraction

Two investigators independently reviewed and extracted information from all publications that met the inclusion criteria. In the case of a conflict, an agreement was reached by discussion between the two reviewers. The following information was sought from each publication: first author’s surname, year of publication, country of origin, ethnicity, cancer type, genotyping method, source of control groups, numbers of cases and controls for each genotype.

### Statistical Analysis

We first assessed the departure of frequencies of miRNA polymorphisms from expectation under Hardy-Weinberg equilibrium (HWE) for each study by using the goodness-of-fit test (chi-square or Fisher exact test) in controls. Crude OR corresponding to 95% CI was used to assess the strength of the association between miRNA polymorphisms and cancer risk according to the methods published by Woolf *et al* [29]. The statistical significance of the pooled OR was determined by the Z-test, and a *P*-value of <0.05 was considered statistically significant. For *miR-146a* G/C, we investigated the association between genetic variants and cancer risk in allelic contrast (C vs. G), homozygote comparisons (CC vs. GG), heterozygote comparisons (GC vs. GG), dominant model (CC+GC vs. GG) and recessive models (CC vs. GC+GG), respectively. The same method was applied to analyze other polymorphisms. Subgroup analyses were also conducted by ethnicity (Caucasian and Asian), cancer types (if one cancer type contained only one individual study, it was combined into other cancer subgroups), source of control (population-based and hospital-based), and sample size (small sample: the total number of controls and cases less than 1000; large sample: the total number of controls and cases not less than 1000).

Statistical heterogeneity between studies was checked by Cocharan’s chi-square based Q-test [30]. However, as the Q test was insensitive in cases where studies were small or few, *I^2^* values were also calculated, which represent the percentage of total variation across studies and provide a result of heterogeneity rather than chance. If the *P*-value for heterogeneity was <0.05, or if *I^2^* was ≥50%, indicating substantial heterogeneity among studies, then a random-effect model using the DerSimonian and Laird method [31], which yielded wider CIs, was chosen to calculate the pooled OR; otherwise, a fixed-effect model using the Mantel-Haenszel method [32] was used. One-way sensitivity analyses were performed to assess the stability of the meta-analysis results [33]. Potential publication bias was estimated using Egger’s linear regression test by visual inspection of the Funnel plot. A *P* value <0.05 was used as an indication of potential publication bias [34]. All statistical analyses were carried out with the STATA software package version 10.0 (Stata Corporation, College Station, TX).

## Results

### Study Identification

**Figure 1:**
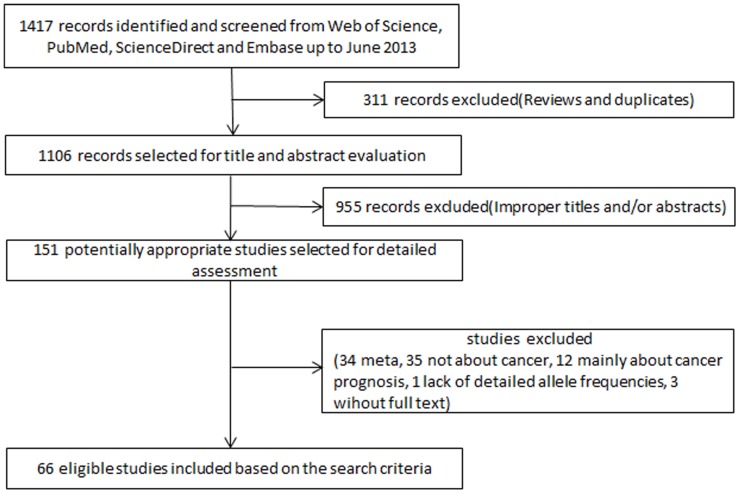
Flow chart of the study selection process.

In total, 66 published articles [15]–[20], [22]–[28], [35]–[87] (Table 1), with 127 comparisons, were identified through literature search with different combinations of key terms and were selected based on the inclusion criteria (Figure 1). During data extraction, 85 out of 151 articles were excluded, including 34 articles on meta-analysis, 35 articles that were not about cancer, 12 articles that were concerned with cancer prognosis, 1 article that provided incomplete polymorphism distribution data, and 3 articles that lacked full text. Two articles [41], [80] that did not provide the distribution of all three genotypes in detail, but presented genotypes as CC+GC and GG were still kept in our analysis. In two studies [70], [85], genotype frequencies were presented separately according to the country of origin of the study subjects, and thus each of these studies was treated as a separate study. In addition, Zhang *et al.* [46] investigated two types of cancers in one study. Each type of cancer in this article was considered separately for meta-analysis.

**Table 1:** Main characteristics of studies included in the meta-analysis.

	Author	Ref	Year	Country	Ethnicity	Cancer type	Design	Genotyping methods	Number of cases/controls	Genotypes distribution of cases/controls			HWE (P)
										miR-146a rs2910164			
										GG	GC	CC	
1	Horikawa	[22]	2008	USA	Caucasian	Renal Cell Cancer	PB	SNPlex assay	261/235	144/126	103/94	14/15	0.65
2	Jazdzewski	[85]	2008	Finland	Caucasian	PTC	PB	SNuPE Assay	206/274	99/150	104/105	3/19	0.91
3	Jazdzewski	[85]	2008	Poland	Caucasian	PTC	PB	SNuPE Assay	201/475	115/286	82/163	4/26	0.66
4	Jazdzewski	[85]	2008	USA	Caucasian	PTC	PB	SNuPE Assay	201/152	91/90	101/52	9/10	0.51
5	Xu	[84]	2008	China	Asian	Liver Cancer	HB	PCR-RFLP	479/504	80/58	241/249	158/197	0.12
6	Yang	[86]	2008	USA	Caucasian	Bladder Cancer	PB	SNPlex assay	691/674	414/385	242/258	35/31	0.14
7	Hoffman	[83]	2009	USA	Caucasian	Breast Cancer	PB/HB	massARRAY	439/478	234/273	176/178	29/27	0.77
8	Hu	[81]	2009	China	Asian	Breast Cancer	PB	PCR-RFLP	1009/1093	165/180	515/551	329/362	0.22
9	Tian	[82]	2009	China	Asian	Lung Cancer	PB	PCR-RFLP	1058/1035	360/364	510/502	188/169	0.85
10	Catucci	[70]	2010	Italy	Caucasian	Breast Cancer	PB	Sequencing	754/1243	409/650	286/520	59/73	0.02
11	Catucci	[70]	2010	Germany	Caucasian	Breast Cancer	PB	Sequencing	805/904	451/536	304/318	50/50	0.75
12	Guo	[66]	2010	China	Asian	ESCC	PB	SNaPshot	444/468	234/206	190/220	20/42	0.12
13	Liu	[71]	2010	USA	Caucasian	SCCHN	HB	PCR-RFLP	1109/1130	630/655	411/405	68/70	0.49
14	Okubo	[20]	2010	Japan	Asian	Gastric Cancer	HB	PCR-RFLP	552/697	73/121	243/322	236/254	0.28
15	Pastrello	[68]	2010	Italy	Caucasian	Mix(breast and ovarian cancer)	PB	Sequencing	101/155	60/90	36/59	5/6	0.33
16	Srivastava	[79]	2010	India	Asian	Gallbladder Cancer	PB	PCR-RFLP	230/224	129/138	90/81	11/5	0.08
17	Xu	[65]	2010	China	Asian	Prostate Cancer	HB	PCR-RFLP	251/280	68/54	135/150	48/76	0.19
18	Zeng	[69]	2010	China	Asian	Gastric Cancer	HB	PCR-RFLP	304/304	62/53	153/132	89/119	0.12
19	Akkiz	[59]	2011	Turkey	Caucasian	Liver Cancer	HB	PCR-RFLP	222/222	137/144	75/67	10/11	0.38
20	Garcia	[56]	2011	French	Caucasian	Breast Cancer	PB	TaqMan	1130/596	676/352	388/220	66/24	0.15
21	George	[62]	2011	India	Asian	Prostate Cancer	PB	PCR-RFLP	159/230	4/7	79/107	76/116	0.00
22	Hishida	[60]	2011	Japan	Asian	Gastric Cancer	HB	PCR-CTPP	583/1637	82/229	271/775	230/633	0.74
23	Mittal	[15]	2011	India	Asian	Bladder Cancer	PB	PCR-RFLP	212/250	127/135	79/108	6/7	0.01
24	Permuth-Wey	[55]	2011	USA	Caucasian	Glioma	PB	GoldenGate	593/614	345/375	198/214	50/25	0.42
25	Vinci	[61]	2011	Italy	Caucasian	NSCLC	NR	HRMA	101/129	44/73	48/45	9/11	0.29
26	Yue	[18]	2011	China	Asian	Cervical Cancer	HB	PCR-RFLP	447/443	118/87	224/206	105/150	0.29
27	Zhang	[58]	2011	China	Asian	Liver Cancer	HB	PIRA–PCR	925/1593	156/291	450/725	319/577	0.02
28	Zhou	[19]	2011	China	Asian	CSCC	HB	PCR-RFLP	226/309	43/34	113/159	70/116	0.06
29	Alshatwi	[40]	2012	Saudi	Asian	Breast Cancer	PB	TaqMan	100/100	2/3	50/46	48/51	0.05
30	Chu	[42]	2012	China	Asian	Oral Cancer	HB	PCR-RFLP	470/425	54/54	242/196	174/175	0.94
31	Hezova	[51]	2012	Czech	Caucasian	Colorectal Cancer	HB	TaqMan	197/212	115/124	70/79	12//9	0.41
32	Kim	[46]	2012	Korea	Asian	Liver Cancer	PB	PCR-RFLP	286/201	27/24	159/103	100/74	0.19
33	Lung	[54]	2012	China	Asian	Nasopharyngeal Carcinoma	PB	Tm-shift	229/3631	24/497	88/1721	117/1413	0.46
34	Mihalache	[47]	2012	Italy and Germany	Caucasian	Cholangiocarcinoma	HB	TaqMan	182/350	118/211	53/122	11/17	0.91
35	Min	[39]	2012	Korea	Asian	Colorectal Cancer	HB	PCR-RFLP	446/502	62/69	233/245	151/188	0.44
36	Wang	[41]	2012	China	Asian	Bladder Cancer	HB	TaqMan	1017/1179	369/340	456/571	192/268	0.34
37	Xiang	[38]	2012	China	Asian	Liver Cancer	HB	PCR-RFLP	100/200	27/45	45/100	28/55	0.97
38	Zhou	[37]	2012	China	Asian	Liver Cancer	PB	PCR-RFLP	186/483	33/71	86/254	67/158	0.06
39	Zhou	[36]	2012	China	Asian	Gastric Cancer	HB	TaqMan	1686/1895	578/551	822/951	286/393	0.64
40	Ma	[32]	2013	China	Asian	TNBC	HB	massARRAY	192/191	35/34	94/93	63/64	0.98
41	Ma	[34]	2013	China	Asian	Colorectal Cancer	HB	TaqMan	1147/1203	444/397	534/614	169/192	0.08
42	ORSÓS	[29]	2013	Hungary	Caucasian	SCCHN	PB	PCR-RFLP	468/468	284/323	168/136	16/9	0.22
43	Song	[35]	2013	USA	Caucasian	OSCC	HB	PCR-RFLP	325/335	184/203	–	–	–
44	Vinci	[33]	2013	Italy	Caucasian	Colorectal Cancer	NR	HRMA	160/178	86/100	57/65	17/13	0.59
45	Wei	[31]	2013	China	Asian	PTC	PB	massARRAY	753/760	136/138	323/345	294/277	0.09
46	Wei	[87]	2013	China	Asian	ESCC	HB	massARRAY	368/370	67/67	184/181	117/122	0.99
47	Yamashita	[30]	2013	Japan	Asian	Malignant melanoma	NR	PCR-RFLP	50/107	0/3	35/53	15/51	0.01
										**miR-196a2 rs11614913**			
										**CC**	**CT**	**TT**	
1	Horikawa	[22]	2008	USA	Caucasian	Renal Cell cancer	PB	SNPlex assay	276/277	105/101	126/117	45/59	0.02
2	Yang	[86]	2008	USA	Caucasian	Bladder Cancer	PB	SNPlex assay	736/731	255/257	348/342	133/132	0.32
3	Hoffman	[83]	2009	USA	Caucasian	Breast Cancer	PB/HB	massARRAY	426/466	181/166	209/229	36/71	0.58
4	Hu	[81]	2009	China	Asian	Breast Cancer	PB	PCR-RFLP	1009/1093	239/218	483/517	287/358	0.21
5	Tian	[82]	2009	China	Asian	Lung Cancer	PB	PCR-RFLP	1058/1035	253/209	512/519	293/307	0.70
6	Catucci	[70]	2010	Italy	Caucasian	Breast Cancer	PB	TaqMan	751/1243	334/532	330/550	87/161	0.32
7	Catucci	[70]	2010	Germany	Caucasian	Breast Cancer	PB	TaqMan	1101/1496	432/584	512/696	157/216	0.71
8	Christensen	[80]	2010	USA	Caucasian	SCCHN	PB	Taqman	484/555	182/188	–	–	–
9	Dou	[77]	2010	China	Asian	Glioma	HB	PCR-LDR	643/656	111/143	343/305	189/208	0.12
10	Kim	[75]	2010	Korea	Asian	Lung Cancer	HB	PCR-FRET	654/640	187/155	305/300	162/185	0.13
11	Li	[67]	2010	China	Asian	Liver Cancer	HB	PCR-RFLP	310/222	78/42	150/102	82/78	0.40
12	Liu	[71]	2010	USA	Caucasian	SCCHN	HB	PCR-RFLP	1109/1130	350/383	565/545	194/202	0.74
13	Okubo	[20]	2010	Japan	Asian	Gastric Cancer	HB	PCR-RFLP	552/697	105/124	281/350	166/223	0.51
14	Peng	[78]	2010	China	Asian	Gastric Cancer	HB	PCR-RFLP	213/213	76/161	94/107	43/50	0.94
15	Qi	[72]	2010	China	Asian	Liver Cancer	HB	PCR-LDR	361/590	82/125	179/304	100/161	0.40
16	Srivastava	[79]	2010	India	Asian	Gallbladder Cancer	PB	PCR-RFLP	230/230	119/136	95/75	16/19	0.07
17	Wang	[76]	2010	China	Asian	ESCC	HB	SNaPshot	458/489	148/128	262/250	48/111	0.60
18	Akkiz	[59]	2011	Turkey	Caucasian	Liver Cancer	HB	PCR-RFLP	185/185	77/58	86/87	22/40	0.49
19	George	[62]	2011	India	Asian	Prostate Cancer	PB	PCR-RFLP	159/230	55/106	101/114	3/10	0.00
20	Hong	[64]	2011	Korea	Asian	Lung Cancer	HB	Taqman	406/428	86/96	224/198	96/134	0.16
21	Jedlinski	[63]	2011	Australia	Caucasian	Breast Cancer	PB	PCR-RFLP	187/171	68/58	86/82	33/31	0.83
22	Mittal	[15]	2011	India	Asian	Bladder Cancer	PB	PCR-RFLP	212/250	76/109	131/127	5/14	0.00
23	Vinci	[61]	2011	Italy	Caucasian	NSCLC	NR	HRMA	101/129	35/58	54/61	12/10	0.27
24	Zhan	[57]	2011	China	Asian	Colorectal Cancer	HB	PCR-RFLP	252/543	68/113	128/267	56/163	0.85
25	Zhang	[58]	2011	China	Asian	Liver Cancer	HB	PIRA–PCR	934/1622	208/328	449/817	277/477	0.52
26	Zhou	[19]	2011	China	Asian	CSCC	HB	PCR-RFLP	226/309	46/58	123/169	57/82	0.08
27	Alshatwi	[40]	2012	Saudi	Asian	Breast Cancer	PB	TaqMan	100/100	35/46	63/50	2/4	0.03
28	Chen	[49]	2012	China	Asian	CRC	HB	PCR–LDR	126/407	27/94	64/206	35/107	0.79
29	Chu	[42]	2012	China	Asian	Oral Cancer	HB	PCR-PFLP	470/425	57/87	277/206	136/132	0.69
30	Hezova	[51]	2012	Czech	Caucasian	Colorectal Cancer	HB	TaqMan	197/212	82/87	89/103	26/22	0.29
31	Kim	[46]	2012	Korea	Asian	Liver Cancer	PB	PCR-RFLP	286/201	58/45	154/107	74/49	0.36
32	Linhares	[52]	2012	Brazil	Caucasian	Breast Cancer	HB	TaqMan	325/274	83/94	148/114	94/66	0.00
33	Min	[39]	2012	Korea	Asian	Colorectal Cancer	HB	PCR-RFLP	446/502	120/100	201/254	125/148	0.63
34	Zhang	[44]	2012	China	Asian	Breast Cancer	PB	PCR-RFLP	248/243	1/17	89/93	148/133	0.89
35	Zhu	[48]	2012	China	Asian	Colorectal Cancer	HB	TaqMan	573/588	140/121	303/295	130/172	0.79
36	Song	[35]	2013	USA	Caucasian	OSCC	HB	PCR-RFLP	325/335	95/96	–	–	–
37	Vinci	[33]	2013	Italy	Caucasian	CRC	NR	HRMA	160/178	62/83	86/84	12/11	0.09
38	Wei	[87]	2013	China	Asian	ESCC	HB	massARRAY	367/370	65/87	196/170	106/113	0.14
										**miR-499 rs3746444**			
										**TT**	**TC**	**CC**	
1	Hu	[81]	2009	China	Asian	Breast Cancer	PB	PCR-RFLP	1093/1009	707/816	258/248	44/29	0.06
2	Tian	[82]	2009	China	Asian	Lung Cancer	PB	PCR-RFLP	1035/1058	781/755	253/254	24/26	0.40
3	Catucci	[70]	2010	Italy	Caucasian	Breast Cancer	PB	Sequencing	1242/756	414/704	295/452	47/86	0.25
4	Catucci	[70]	2010	Germany	Caucasian	Breast Cancer	PB	Sequencing	925/823	536/601	250/290	37/34	0.89
5	Liu	[71]	2010	USA	Caucasian	SCCHN	HB	PCR-RFLP	1130/1109	745/710	309/366	55/54	0.44
6	Okubo	[20]	2010	Japan	Asian	Gastric Cancer	HB	PCR-RFLP	697/552	364/466	151/198	37/33	0.05
7	Srivastava	[79]	2010	India	Asian	Gallbladder Cancer	PB	PCR-RFLP	230/230	112/121	97/94	21/15	0.57
8	Akkiz	[59]	2011	Turkey	Caucasian	Liver Cancer	HB	PCR-RFLP	222/222	45/47	87/93	90/82	0.04
9	George	[62]	2011	India	Asian	Prostate Cancer	PB	PCR-RFLP	230/159	48/104	98/92	13/34	0.07
10	Mittal	[15]	2011	India	Asian	Bladder Cancer	PB	PCR-RFLP	250/212	95/121	92/94	25/35	0.02
11	Vinci	[61]	2011	Italy	Caucasian	Lung Cancer	NR	HRMA	129/101	53/70	41/48	7/11	0.50
12	Zhou	[19]	2011	China	Asian	CSCC	HB	PCR-RFLP	309/226	134/223	84/71	8/15	0.00
13	Alshatwi	[40]	2012	Saudi	Asian	Breast Cancer	PB	TaqMan	100/100	30/45	62/40	8/15	0.23
14	Chu	[42]	2012	China	Asian	Oral Cancer	HB	PCR-PFLP	425/270	339/356	119/66	12/3	0.98
15	Kim	[46]	2012	Korea	Asian	Liver Cancer	PB	PCR-RFLP	201/286	200/120	81/74	5/7	0.28
16	Min	[39]	2012	Korea	Asian	Colorectal Cancer	HB	PCR-RFLP	502/446	292/334	142/154	12/14	0.45
17	Xiang	[38]	2012	China	Asian	Liver Cancer	HB	PCR-RFLP	200/100	36/106	40/71	24/23	0.04
18	Zhou	[37]	2012	China	Asian	Liver Cancer	PB	PCR-RFLP	483/186	141/371	41/100	4/12	0.10
19	Song	[35]	2013	USA	Caucasian	OSCC	HB	PCR-RFLP	325/335	184/214	–	–	–
20	Vinci	[33]	2013	Italy	Caucasian	CRC	NR	HRMA	178/160	93/105	32/56	35/17	0.03
21	Wei	[87]	2013	China	Asian	ESCC	HB	massARRAY	358/376	291/289	60/76	7/11	0.14
										**miR-149 rs2292832**			
										**CC**	**CT**	**TT**	
1	Hu	[81]	2009	China	Asian	Breast Cancer	PB	PCR-RFLP	1009/1093	450/482	460/503	99/108	0.16
2	Tian	[82]	2009	China	Asian	Lung Cancer	PB	PCR-RFLP	1058/1035	123/112	472/453	463/470	0.86
3	Liu	[71]	2010	USA	Caucasian	SCCHN	HB	PCR-RFLP	1109/1130	580/586	441/445	88/99	0.27
4	Vinci	[61]	2011	Italy	Caucasian	NSCLC	NR	HRMA	101/129	44/65	41/53	16/11	0.97
5	Chu	[42]	2012	China	Asian	Oral Cancer	HB	PCR-PFLP	470/425	37/26	88/84	345/315	0.00
6	Kim	[46]	2012	Korea	Asian	Liver Cancer	PB	PCR-RFLP	286/201	24/21	113/97	149/83	0.34
7	Min	[39]	2012	Korea	Asian	Colorectal Cancer	HB	PCR-RFLP	446/502	48/51	177/219	221/232	0.95
8	Zhang	[43]	2012	China	Asian	Colorectal Cancer	PB	PCR-RFLP	443/435	50/46	190/202	203/187	0.43
9	Zhang	[43]	2012	China	Asian	Gastric Cancer	PB	PCR-RFLP	274/269	41/35	101/120	132/114	0.70
10	Zhang	[44]	2012	China	Asian	Breast Cancer	PB	PCR-RFLP	245/229	23/24	102/113	120/92	0.21
11	Song	[35]	2013	USA	Caucasian	OSCC	HB	PCR-RFLP	325/335	158/162	–	–	–
12	Vinci	[33]	2013	Italy	Caucasian	CRC	NR	HRMA	160/178	79/86	58/75	23/17	0.91
										**miR-27a rs895919**			
										**AA**	**AG**	**GG**	
1	Hoffman	[83]	2009	USA	Mixed	Breast Cancer	PB/HB	massARRAY	434/477	184/220	200/211	50/46	0.65
2	Sun	[73]	2010	China	Asian	Gastric Cancer	HB	PCR-RFLP	304/304	115/145	135/119	54/40	0.05
3	Yang	[74]	2010	Germany	Caucasian	Breast Cancer	PB	Sequencing	1189/1416	576/605	486/660	127/151	0.14
4	Catucci	[53]	2012	Italy	Caucasian	Breast Cancer	PB	TaqMan	1025/1593	547/803	388/633	90/157	0.05
5	Hezova	[51]	2012	Czech	Caucasian	Colorectal Cancer	HB	TaqMan	197/212	88/93	86/94	23/25	0.29
6	Shi	[45]	2012	China	Asian	Renal Cell Cancer	HB	TaqMan	594/600	334/288	213/262	47/50	0.37
7	Zhang	[44]	2012	China	Asian	Breast Cancer	PB	PCR-RFLP	245/243	60/75	144/109	41/59	0.12
8	Zhou	[50]	2012	China	Asian	Gastric Cancer	HB	massARRAY	295/413	166/214	122/167	7/32	0.94
9	Wei	[87]	2013	China	Asian	ESCC	HB	massARRAY	379/377	216/208	143/139	20/30	0.14

HB: hospital based; PB: population based; Mixed: hospital and population based; NR: not reported; PTC: papillary thyroid carcinoma; ESCC: esophageal squamous cell carcinoma; SCCHN: squamous cell carcinoma of the head and neck; NSCLC: non-small cell lung cancer; CSCC: cervical cancer; TNBC: triple negative breast cancer; OSCC: oral squamous cell carcinoma; CRC: colorectal cancer; PCR-RFLP: polymerase chain reaction–restriction fragment length polymorphism; HRMA: high-resolution melting analysis; PIRA–PCR: primer-introduced restriction analysis-polymerase chain reaction; PCR-LDR: polymerase chain reaction-ligation detection reaction; PCR-FRET: polymerase chain reaction-fluorescence resonance energy transfer; Tm-shift: melting-temperature –shift allele-specific genotyping; HWE: Hardy-Weinberg equilibrium; *P*: p value.

Overall, 47, 38, 21, 12, and 9 studies were pooled for meta-analysis of the rs2910164, rs11614913, rs3746444, rs2292832, and rs895919, respectively. Among all the included articles, there were 11 articles on liver cancer and breast cancer each, 8 studies on gastric cancer and colorectal cancer each, 5 studies on squamous cell carcinoma of the head and neck (SCCHN), 4 studies on lung cancer, 3 studies on bladder cancer and esophageal squamous cell carcinoma (ESCC) each, 2 studies on prostate cancer, glioma cancer, renal cell cancer, papillary thyroid carcinoma (PTC) and cervical cancer each, and 1 study each on gallbladder cancer, malignant melanoma and breast/ovarian cancer. The ethnicity of subjects in 42 studies and 24 studies were Asian and Caucasian, respectively. The controls from 37 studies came from a hospital-based population, whereas 25 studies had population-based controls. One study included both population-based and hospital-based controls [83], while three studies lacked the information of control source [36], [39], [61]. To determine the SNPs, multiple genotyping methods were employed including polymerase chain reaction-restriction fragment length polymorphism (PCR-RFLP), TaqMan assay, SNPlex, SNuPE Assay, high-resolution melting analysis (HRMA), polymerase chain reaction-ligation detection reaction (PCR-LDR), direct sequencing, SNaPshot, Sequenom’s MassARRAY, fluorescence labeled hybridization (PCR-FRET), polymerase chain reaction with confronting two-pair primers (PCR-CTTP), Illumina’s GoldenGate, primer introduced restriction analysis- polymerase chain reaction (PIRA-PCR) and Tm-shift allele-specific genotyping. Genotypic distribution of most of the studied SNPs was in agreement with HWE (*P*>0.05) in controls.

### Quantitative Synthesis

#### miR-146a rs2910164

For miR-146a rs2910164 polymorphism, our study contained 47 comparisons with 22,055 cases and 29,138 controls. The frequency of the rs2910164 C allele had a significantly higher representation in the Asian population compared to the Caucasian population (Asian: 54.3%, 95% CI = 49.1–59.4%; Caucasian: 24.2%, 95% CI = 22.9–25.4%; *P*<0.001).

The results of the meta-analysis on rs2910164 and cancer risk are shown in Table 2. Overall, no significant association was found between rs2910164 and cancer risk under any genetic model when all the eligible studies were pooled into the meta-analysis. After exclusion of four studies [15], [36], [58], [70], whose genotypic distributions in controls were not in agreement with HWE, the results did not significantly change.

**Table 2:** Meta-analysis of miR-146a rs2910164 polymorphism with cancer risk.

Variables	n^a^	C vs. G				CC vs. GG				GC vs. GG				CC+GC vs. GG				CC vs. GC+GG			
		OR(95% CI)	*P*	*P-H*	*I^2^*	OR(95% CI)	*P*	*P-H*	*I^2^*	OR(95% CI)	*P*	*P-H*	*I^2^*	OR(95% CI)	*P*	*P-H*	*I^2^*	OR(95% CI)	*P*	*P-H*	*I^2^*
**Total**	47	0.978(0.931–1.027)	0.375	<0.001	63.6	0.952(0.851–1.065)	0.393	<0.001	60.5	0.982(0.921–1.048)	0.588	<0.001	45.9	0.983(0.919–1.051)	0.614	<0.001	55.4	0.959(0.880–1.045)	0.339	<0.001	58.9
**Cancer type**																					
Bladder Cancer	3	0.838(0.762–0.921)	0.001	0.324	11.2	0.724(0.587–0.893)	0.003	0.241	29.7	0.789(0.689–0.904)	0.001	0.526	0.0	0.781(0.687–0.889)	<0.001	0.290	19.3	0.836(0.693–1.010)	0.063	0.446	0.0
Breast Cancer	7	1.032(0.966–1.102)	0.353	0.864	0.0	1.138(0.970–1.335)	0.112	0.818	0.0	0.999(0.907–1.099)	0.976	0.485	0.0	1.025(0.935–1.123)	0.601	0.682	0.0	1.073(0.944–1.219)	0.282	0.504	0.0
Cervical Cancer	2	0.719(0.620–0.835)	<0.001	0.796	0.0	0.503(0.370–0.684)	<0.001	0.814	0.0	0.721(0.545–0.953)	0.022	0.254	23.1	0.632(0.485–0.823)	0.001	0.382	0.0	0.654(0.520–0.822)	<0.001	0.359	0.0
Colorectal Cancer	4	0.912(0.833–0.999)	0.047	0.324	13.6	0.873(0.716–1.064)	0.179	0.281	21.5	0.854(0.740–0.985)	0.030	0.376	3.4	0.859(0.750–0.984)	0.028	0.294	19.2	0.926(0.785–1.091)	0.357	0.393	0.0
ESCC	2	0.841(0.631–1.121)	0.237	0.047	74.6	0.648(0.288–1.457)	0.294	0.021	81.1	0.834(0.667–1.042)	0.109	0.235	29.2	0.815(0.585–1.134)	0.224	0.142	53.7	0.700(0.360–1.362)	0.294	0.033	77.9
Gastric Cancer	4	0.953(0.782–1.162)	0.633	<0.001	86.4	0.915(0.625–1.339)	0.648	<0.001	84.1	0.907(0.806–1.020)	0.104	0.136	45.8	0.960(0.742–1.240)	0.753	0.011	73.1	0.919(0.700–1.206)	0.543	<0.001	83.5
Lung Cancer	2	1.079(0.959–1.214)	0.205	0.209	36.7	1.139(0.891–1.455)	0.300	0.710	0.0	1.264(0.753–2.122)	0.375	0.068	69.9	1.246(0.799–1.945)	0.332	0.095	64.2	1.104(0.885–1.377)	0.381	0.912	0.0
Primary Liver Cancer	7	0.950(0.879–1.027)	0.199	0.444	0.0	0.919(0.778–1.086)	0.320	0.313	15.3	0.969(0.840–1.118)	0.666	0.103	43.2	0.951(0.831–1.088)	0.463	0.139	38.0	0.924(0.820–1.040)	0.191	0.641	0.0
Prostate Cancer	2	0.801(0.660–0.971)	0.024	0.200	39.1	0.565(0.354–0.900)	0.016	0.234	29.5	0.761(0.509–1.137)	0.182	0.384	0.0	0.685(0.466–1.007)	0.054	0.340	0.0	0.757(0.568–1.008)	0.057	0.235	29.1
PTC	4	1.070(0.958–1.196)	0.230	0.520	0.0	0.639(0.321–1.272)	0.202	0.040	63.9	1.319(0.985–1.768)	0.063	0.042	63.4	1.189(1.009–1.402)	0.039	0.164	41.2	0.547(0.244–1.227)	0.143	0.006	75.6
SCCHN	5	1.160(0.956–1.407)	0.133	0.005	76.3	1.223(0.981–1.526)	0.074	0.134	46.3	1.147(1.003–1.311)	0.045	0.366	5.3	1.165(1.035–1.310)	0.011	0.349	10.0	1.187(0.807–1.744)	0.384	0.003	78.4
other	5	1.103(0.969–1.255)	0.136	0.173	37.3	1.673(1.163–2.408)	0.006	0.285	20.4	1.026(0.867–1.215)	0.763	0.758	0.0	1.093(0.930–1.285)	0.281	0.705	0.0	1.174(0.603–2.285)	0.638	0.007	71.8
**Ethnicity**																					
Caucasian	19	1.069(1.015–1.126)	0.011	0.476	0.0	1.183(1.030–1.359)	0.017	0.193	22.0	1.077(0.978–1.185)	0.131	0.017	46.2	1.074(1.009–1.142)	0.024	0.135	27.0	1.162(1.014–1.331)	0.030	0.076	34.4
Asian	28	0.926(0.870–0.986)	0.017	<0.001	68.5	0.868(0.762–0.989)	0.033	<0.001	64.2	0.899(0.848–0.953)	<0.001	0.06	31.3	0.899(0.822–0.983)	0.020	0.001	52.5	0.907(0.827–0.995)	0.039	0.001	62.9
**Design**																					
HB	21	0.903(0.845–0.964)	0.002	<0.001	62.8	0.821(0.716–0.942)	0.005	<0.001	60.3	0.908(0.829–0.995)	0.038	0.026	41.8	0.893(0.809–0.986)	0.025	0.001	57.4	0.869(0.795–0.951)	0.002	0.015	45.4
PB	22	1.046(0.983–1.113)	0.159	0.010	46.0	1.108(0.940–1.306)	0.223	0.008	47.1	1.027(0.941–1.122)	0.548	0.023	41.4	1.044(0.961–1.134)	0.307	0.033	39.0	1.087(0.940–1.256)	0.261	0.001	56.4
**Sample size**																					
≥1000	16	1.015(0.947–1.088)	0.676	<0.001	75.0	1.074(0.924–1.247)	0.352	<0.001	73.1	0.949(0.880–1.022)	0.167	0.024	45.6	0.976(0.895–1.064)	0.583	<0.001	63.6	1.085(0.967–1.218)	0.164	0.001	69.8
<1000	31	0.947(0.882–1.016)	0.128	0.001	51.6	0.836(0.712–0.982)	0.029	0.010	41.3	1.015(0.913–1.129)	0.783	0.005	44.9	0.993(0.897–1.100)	0.898	0.001	50.4	0.841(0.752–0.940)	0.002	0.042	33.1
**HWE**																					
Yes	42	0.982(0.931–1.037)	0.517	<0.001	66.1	0.940(0.833–1.060)	0.315	<0.001	62.4	0.986(0.920–1.056)	0.683	0.001	47.0	0.981(0.912–1.057)	0.619	<0.001	58.2	0.959(0.875–1.051)	0.368	<0.001	59.7

ESCC: esophageal squamous cell carcinoma; PTC: papillary thyroid carcinoma; SCCHN: squamous cell carcinoma of the head and neck; HB: hospital based; PB: population based; HWE: Hardy-Weinberg equilibrium; OR: odds ratio; CI: confidence interval; *P*: p value; *P-H*:P value of Q for heterogeneity test; *I^2^*: 0–25%, no heterogeneity; 25–50%, modest heterogeneity; 50%, high heterogeneity;

a Number of studies involved Random effects model was used when P value of Q for heterogeneity test (P-H)<0.05 or *I^2^*>50%; otherwise, fixed effect model was used.

However, in the stratified analysis by cancer type, the C allele and CC genotype of rs2910164 were found to be associated with an inverse risk of bladder cancer under all genetic models, except for the recessive model (C vs. G: OR = 0.838, 95% CI = 0.762–0.921, *P*_H = _0.324; CC vs. GG: OR = 0.724, 95% CI = 0.587–0.893, *P*_H = _0.241; GC vs. GG: OR = 0.789, 95% CI = 0.689–0.904, *P*_H = _0.526; CC+GC vs. GG: OR = 0.781, 95% CI = 0.687–0.889, *P*_H_ <0.290), cervical cancer under all genetic models (C vs. G: OR = 0.719, 95% CI = 0.620–0.839, *P*_H = _0.796; CC vs. GG: OR = 0.503, 95% CI = 0.370–0.684, *P*_H = _0.814; GC vs. GG: OR = 0.721, 95% CI = 0.545–0.953, *P*_H = _0.254; CC+GC vs. GG: OR = 0.632, 95% CI = 0.485–0.823, *P*_H = _0.382; CC vs. GC+GG: OR = 0.654, 95% CI = 0.520–0.822, *P*_H = _0.359), colorectal cancer under allelic contrast, heterozygote comparison and the dominant model (C vs. G: OR = 0.912, 95% CI = 0.833–0.999, *P*_H = _0.324; GC vs. GG: OR = 0.854, 95% CI = 0.740–0.985, *P*_H = _0.376; CC+GC vs. GG: OR = 0.859, 95% CI = 0.750–0.984, *P*_H = _0.294) and prostate cancer under allelic contrast and homozygote comparison (C vs. G: OR = 0.801, 95% CI = 0.660–0.971, *P*_H = _0.200; CC vs. GG: OR = 0.565, 95% CI = 0.354–0.900, *P*_H = _0.234). In addition, rs2910164 was found to be associated with risks of PTC and SCCHN in the heterozygote comparison (CC+GC vs. GG: OR = 1.189, 95% CI = 1.009–1.402, *P*_H = _0.164) and the dominant model (GC vs. GG: OR = 1.147, 95% CI = 1.003–1.311, *P*_H = _0.366). Nevertheless, the direction of ORs in the two cancers was opposite to that of the former four cancers.

When stratified analysis was performed by ethnicity of study population, rs2910164 C allele and CC genotype were shown to be associated with substantial decrease in cancer risk in Asian populations under all genetic models. On the contrary, Caucasian C or CC carriers were more susceptible to cancers under all genetic models, except for heterozygote comparison. Further subgroup analysis revealed the C allele or CC genotype to be associated with decreased cancer risk in studies of hospital-based study design for all genetic models, but not in studies of population based study design. When stratified on the basis of sample size, the CC genotype had an effect of decreased cancer risk among small size subgroups compared with GG genotype or G allele carriers.

#### miR-196a2 rs11614913

The miR-196a2 rs11614913 polymorphism was analyzed in 38 comparisons with 16,414 cases and 19,465 controls. We also observed a wide variation of the T allele frequency across different ethnicities (Asian: 49.8%, 95% CI = 45.3%–54.3%; Caucasian: 38.8%, 95% CI = 35.9%–41.7%; *P = *0.002).

Table 3 summarizes the results from the meta-analysis of miR-196a2 rs11614913 and cancer risk. In the overall analysis, we found a significant association between rs11614913 and reduced cancer risk in the allelic contrast (OR = 0.949, 95% CI = 0.902–0.998, *P*_H_ <0.001), homozygote comparison (OR = 0.861, 95% CI = 0.772–0.959 *P*_H_<0.001) and recessive model (OR = 0.865, 95% CI = 0.802–0.934, *P*_H = _0.002). Removing four studies with genotype frequencies in controls that deviated from HWE did not alter the pooled results [15], [43], [53], [62].

**Table 3:** Meta-analysis of miR-196a2 rs11614913 polymorphism with cancer risk.

Variables	n^a^	T vs. C				TT vs. CC				CT vs. CC				TT+CT vs. CC				TT vs. CT+CC			
		OR(95% CI)	*P*	*P-H*	*I^2^*	OR(95% CI)	*P*	*P-H*	*I^2^*	OR(95% CI)	*P*	*P-H*	*I^2^*	OR(95% CI)	*P*	*P-H*	*I^2^*	OR(95% CI)	*P*	*P-H*	*I^2^*
**Total**	38	0.949(0.902–0.998)	0.044	<0.001	58.2	0.861(0.772–0.959)	0.007	<0.001	58.7	1.033(0.951–1.123)	0.441	<0.001	56.6	0.984(0.909–1.065)	0.685	<0.001	60.0	0.865(0.802–0.934)	<0.001	0.002	45.2
**Cancer type**																					
Bladder Cancer	2	1.032(0.906–1.174)	0.639	0.562	0.0	0.961(0.724–1.277)	0.786	0.224	32.4	1.192(0.837–1.696)	0.331	0.106	61.7	1.102(0.915–1.327)	0.307	0.171	46.5	0.738(0.320–1.701)	0.476	0.100	63.1
Breast Cancer	8	0.978(0.868–1.102)	0.716	0.002	68.3	0.903(0.699–1.167)	0.436	0.004	66.9	0.976(0.888–1.074)	0.623	0.12	38.9	0.989(0.841–1.164)	0.898	0.014	60.3	0.915(0.765–1.095)	0.334	0.031	54.5
Colorectal Cancer	6	0.910(0.794–1.043)	0.177	0.061	52.6	0.754(0.627–0.907)	0.003	0.108	44.6	0.878(0.755–1.021)	0.091	0.168	35.9	0.848(0.735–0.979)	0.025	0.082	48.9	0.838(0.721–0.974)	0.021	0.165	36.3
ESCC	2	0.863(0.551–1.351)	0.518	0.001	90.6	0.685(0.209–2.245)	0.532	<0.001	93.9	1.166(0.692–1.962)	0.564	0.030	78.7	1.020(0.537–1.935)	0.953	0.005	87.3	0.610(0.268–1.390)	0.240	0.001	91.4
Gastric Cancer	2	0.893(0.778–1.024)	0.104	0.230	30.5	0.803(0.608–1.062)	0.125	0.306	4.5	0.839(0.653–1.077)	0.167	0.163	48.5	0.819(0.647–1.037)	0.097	0.162	48.8	0.894(0.722–1.107)	0.305	0.698	0.0
Lung Cancer	4	0.893(0.821–0.971)	0.008	0.149	43.8	0.794(0.672–0.938)	0.007	0.259	25.5	0.991(0.771–1.274)	0.945	0.059	59.7	0.935(0.745–1.175)	0.565	0.075	56.6	0.842(0.737–0.962)	0.011	0.201	0.201
Primary Liver Cancer	5	0.890(0.767–1.032)	0.123	0.034	61.7	0.790(0.589–1.061)	0.117	0.041	59.8	0.873(0.754–1.010)	0.068	0.776	0.0	0.859(0.748–0.986)	0.030	0.334	12.5	0.871(0.690–1.100)	0.248	0.043	59.4
SCCHN	4	1.067(0.965–1.179)	0.205	0.442	0.0	1.241(0.841–1.831)	0.276	0.099	63.3	1.490(0.835–2.658)	0.177	0.006	86.7	1.123(0.851–1.481)	0.413	0.006	76.1	0.948(0.797–1.127)	0.544	0.683	0.0
other	5	1.026(0.928–1.135)	0.613	0.352	9.5	0.966(0.776–1.201)	0.754	0.491	0.0	1.306(1.106–1.542)	0.002	0.188	34.9	1.212(1.035–1.419)	0.017	0.159	39.3	0.853(0.716–1.017)	0.076	0.720	0.0
**Ethnicity**																					
Caucasian	14	0.981(0.894–1.076)	0.683	0.002	61.8	0.934(0.766–1.138)	0.496	0.003	61.7	1.023(0.946–1.108)	0.565	0.3	14.7	0.989(0.895–1.092)	0.825	0.048	42.3	0.918(0.788–1.070)	0.276	0.035	47.1
Asian	24	0.934(0.879–0.991)	0.025	0.001	55.0	0.827(0.727–0.940)	0.004	0.001	55.5	1.043(0.924–1.177)	0.500	<0.001	65.9	0.986(0.878–1.107)	0.808	<0.001	66.6	0.845(0.773–0.923)	<0.001	0.011	44.1
**Design**																					
HB	21	0.918(0.855–0.986)	0.019	<0.001	64.5	0.849(0.726–0.993)	0.040	<0.001	69.5	0.997(0.881–1.127)	0.956	<0.001	63.4	0.946(0.842–1.064)	0.355	<0.001	65.6	0.848(0.763–0.942)	0.002	0.001	56.4
PB	14	0.963(0.916–1.012)	0.132	0.152	29.2	0.869(0.783–0.966)	0.009	0.553	0.0	1.064(0.943–1.201)	0.314	0.024	48.9	1.011(0.907–1.127)	0.843	0.022	48.5	0.908(0.832–0.991)	0.031	0.525	0.0
**Sample size**																					
≥1000	11	0.941(0.904–0.979)	0.003	0.131	33.5	0.880(0.811–0.955)	0.002	0.143	32.0	0.964(0.902–1.031)	0.287	0.085	39.6	0.936(0.857–1.021)	0.137	0.048	45.8	0.904(0.845–0.966)	0.003	0.593	0.0
<1000	27	0.966(0.889–1.050)	0.416	<0.001	65.0	0.854(0.709–1.029)	0.098	<0.001	65.4	1.092(0.959–1.243)	0.183	<0.001	60.3	0.835(0.769–0.907)	<0.001	<0.001	64.4	0.833(0.729–0.952)	0.007	<0.001	55.3
**HWE**																					
Yes	32	0.929(0.884–0.977)	0.004	<0.001	55.1	0.851(0.763–0.948)	0.003	<0.001	58.7	0.990(0.914–1.073)	0.815	0.001	50.9	0.948(0.874–1.028)	0.196	<0.001	56.8	0.863(0.800–0.931)	<0.001	0.004	44.7

ESCC: esophageal squamous cell carcinoma; SCCHN: squamous cell carcinoma of the head and neck; HB: hospital based; PB: population based; HWE: Hardy-Weinberg equilibrium; OR: odds ratio; CI: confidence interval; *P*: p value; *P-H*: P value of Q for heterogeneity test; *I^2^*: 0–25%, no heterogeneity; 25–50%, modest heterogeneity; 50%, high heterogeneity;

a Number of studies involved.

Random effects model was used when P value of Q for heterogeneity test (P-H) <0.05 or *I^2^*>50%; otherwise, fixed effect model was used.

In subgroup analysis by cancer type, significant association between rs11614913 and decreased cancer risk was found for lung cancer (T vs. C: OR = 0.893, 95% CI = 0.821–0.971, *P*_H = _0.149; TT vs. CC: OR = 0.794, 95% CI = 0.627–0.938, *P*_H = _0.259; TT vs. CT+CC: OR = 0.842, 95% CI = 0.737–0.962, *P*_H = _0.201) and colorectal cancer (TT vs. CC: OR = 0.754, 95% CI = 0.627–0.907, *P*_H = _0.108; TT+CT vs. CC: OR = 0.848, 95% CI = 0.735–0.979, *P*_H = _0.082; TT vs. CT+CC: OR = 0.838, 95% CI = 0.721–0.974, *P*_H = _0.165). For liver cancer, T allele carriers showed decreased cancer susceptibility compared with homozygote CC (OR = 0.859, 95% CI = 0.748–0.986, *P*_H = _0.334). However, no association was found between rs11614913 and bladder cancer, breast cancer, ESCC, gastric cancer, or SCCHN.

In ethnic subgroup analysis, a strong association was found between rs11614913 and cancer risk in the allelic contrast, the homozygote comparison, and the recessive model among Asians, whereas negative results were obtained for Caucasians in all genetic models. With respect to the control source, decreased risk was observed in both the hospital- and population-based controls for the homozygote comparison and the recessive model. We also found a reduced risk for allelic contrast in hospital-based studies. In stratified analysis by sample size, significant association of decreased cancer risk was found in both of the subgroups.

#### miR-499 rs3746444

For miR-499 rs3746444, 21 comparisons with 8,888 cases and 10,292 controls were included. No significant difference in C allele frequency between Asians and Caucasians was observed (Asian: 22.2%, 95% CI = 16.7%–27.7%; Caucasian: 29.9%, 95% CI = 14.4%–45.4%; *P = *0.178).

The results of the meta-analysis for miR-499 rs3746444 and the risk of cancer are presented in Table 4. Overall, we observed that rs3746444 could decrease the cancer risk in the allelic contrast (OR = 1.106, 95% CI = 1.005–1.218, *P*_H_ <0.001) and the dominant model (OR = 1.148, 95% CI = 1.020–1.292, *P*_H_ <0.001). However, this association disappeared after the exclusion of six studies [15], [35], [40], [52], [62], [80], whose genotypic distribution in controls was derived from HWE.

**Table 4:** Meta-analysis of miR-499 rs3746444 polymorphism with cancer risk.

Variables	n^a^	C vs. T				CC vs. TT				TC vs. TT				CC+TC vs. TT				CC vs. TC+TT			
		OR(95% CI)	*P*	*P-H*	*I^2^*	OR(95% CI)	*P*	*P-H*	*I^2^*	OR(95% CI)	*P*	*P-H*	*I^2^*	OR(95% CI)	*P*	*P-H*	*I^2^*	OR(95% CI)	*P*	*P-H*	*I^2^*
**Total**	21	1.106(1.005–1.218)	0.040	<0.001	67.2	1.167(0.969–1.405)	0.103	0.042	38.3	1.126(0.985–1.288)	0.081	<0.001	71.9	1.148(1.020–1.292)	0.022	<0.001	69.0	1.100(0.903–1.339)	0.344	0.007	49.4
**Cancer type**																					
Breast Cancer	4	1.101(1.006–1.204)	0.036	0.214	33.0	1.165(0.915–1.482)	0.215	0.189	37.1	1.163(0.952–1.420)	0.140	0.047	62.3	1.150(0.973–1.359)	0.102	0.102	51.7	1.065(0.712–1.595)	0.758	0.059	59.8
Colorectal Cancer	2	1.136(0.938–1.375)	0.192	0.161	49.1	1.557(0.670–3.621)	0.304	0.096	63.9	0.867(0.541–1.390)	0.554	0.100	63.0	1.045(0.831–1.314)	0.705	0.964	0.0	1.645(0.611–4.428)	0.325	0.047	74.6
Lung Cancer	2	0.963(0.822–1.129)	0.643	0.828	0.0	0.880(0.538–1.439)	0.610	0.919	0.0	0.981(0.812–1.185)	0.843	0.595	0.0	0.970(0.809–1.163)	0.742	0.682	0.0	0.874(0.537–1.424)	0.589	0.836	0.0
Primary Liver Cancer	4	1.094(0.737–1.623)	0.656	<0.001	83.3	1.187(0.560–2.516)	0.655	0.017	70.5	1.007(0.696–1.458)	0.970	0.048	62.1	1.074(0.685–1.683)	0.757	0.004	77.2	1.201(0.675–2.136)	0.533	0.065	58.6
SCCHN	3	1.290(0.593–2.804)	0.521	<0.001	95.4	1.774(0.429–7.328)	0.429	0.030	78.6	1.220(0.527–2.821)	0.643	<0.001	94.8	1.289(0.751–2.215)	0.357	<0.001	91.9	1.685(0.503–5.643)	0.398	0.061	71.5
other	6	1.103(0.988–1.231)	0.081	0.124	42.2	1.078(0.823–1.413)	0.585	0.494	0.0	1.280(0.935–1.753)	0.123	<0.001	77.7	1.223(0.947–1.579)	0.124	0.006	69.7	0.946(0.730–1.226)	0.675	0.121	42.6
**Ethnicity**																					
Caucasian	7	1.003(0.926–1.086)	0.951	0.197	31.8	1.110(0.912–1.352)	0.299	0.233	26.9	0.939(0.846–1.042)	0.236	0.143	39.4	0.997(0.908–1.095)	0.952	0.166	34.3	1.139(0.948–1.368)	0.166	0.088	47.8
Asian	14	1.142(0.999–1.305)	0.052	<0.001	71.5	1.169(0.894–1.529)	0.253	0.036	44.7	1.234(1.035–1.471)	0.019	<0.001	73.9	1.220(1.032–1.442)	0.020	<0.001	73.7	1.039(0.781–1.381)	0.794	0.009	53.4
**Design**																					
HB	9	1.188(0.962–1.465)	0.109	<0.001	82.6	1.283(0.910–1.809)	0.156	0.036	53.3	1.163(0.899–1.504)	0.250	<0.001	80.5	1.216(0.965–1.533)	0.097	<0.001	80.7	1.224(1.004–1.491)	0.045	0.101	39.2
PB	10	1.055(0.985–1.130)	0.127	0.088	40.4	1.061(0.881–1.278)	0.533	0.369	7.9	1.142(0.969–1.346)	0.114	0.001	67.4	1.110(0.961–1.282)	0.156	0.006	60.8	0.968(0.808–1.160)	0.726	0.097	41.5
**Sample size**																					
≥1000	6	1.031(0.930–1.143)	0.560	0.039	57.4	1.135(0.946–1.362)	0.173	0.259	23.3	0.992(0.913–1.077)	0.840	0.076	49.9	1.015(0.901–1.142)	0.809	0.049	55.1	1.135(0.948–1.359)	0.167	0.293	18.5
<1000	15	1.157(0.996–1.343)	0.056	<0.001	68.7	1.165(0.869–1.562)	0.307	0.030	46.1	1.225(0.985–1.523)	0.068	<0.001	73.7	1.241(1.038–1.485)	0.018	<0.001	68.5	1.043(0.762–1.429)	0.791	0.003	58.5
**HWE**																					
Yes	14	1.049(0.939–1.172)	0.397	<0.001	67.8	1.063(0.902–1.252)	0.465	0.275	16.4	1.109(0.952–1.293)	0.184	<0.001	74.0	1.094(0.947–1.265)	0.222	<0.001	73.2	1.006(0.857–1.181)	0.938	0.111	33.0

SCCHN: squamous cell carcinoma of the head and neck; HB: hospital based; PB: population based; HWE: Hardy-Weinberg equilibrium; OR: odds ratio; CI: confidence interval; *P*: p value; *P-H*: P value of Q for heterogeneity test; *I^2^*: 0–25%, no heterogeneity; 25–50%, modest heterogeneity; 50%, high heterogeneity;

a Number of studies involved.

Random effects model was used when P value of Q for heterogeneity test (P-H)<0.05 or *I^2^*>50%; otherwise, fixed effect model was used.

In stratified analysis by cancer type, significant associations were only maintained in breast cancer under allelic contrast (OR = 1.101, 95% CI = 1.006–1.204, *P*_H = _0.214), but no significant association was observed with colorectal cancer, lung cancer, liver cancer, SCCHN, and other cancers under any genetic model. Subgroup analysis by ethnicity showed a decreased cancer risk in the Asian population (TC vs. TT: OR = 1.234, 95% CI = 1.035–1.471, *P*_H_ <0.001; TC+CC vs. TT: OR = 1.220, 95% CI = 1.032–1.442, *P*_H_ <0.001), but not in the Caucasian population. Based on study design, studies with hospital-based controls showed elevated risk (CC vs. TC+TT: OR = 1.224, 95% CI = 1.004–1.491, *P*_H_ = 0.045). However, studies with population-based controls showed no significant association. Further subgroup analysis by sample size revealed increased cancer risks only in a small sample group using the dominant model (TC+CC vs. TT: OR = 1.241, 95% CI = 1.038–1.485, *P*_H_ <0.001).

#### miR-149 rs2292832

Twelve comparisons with 5926 cases and 5961 controls assessed for the association between miR-149 rs2292832 polymorphism and cancer risk. The frequency of T allele was significant higher in Asian population compared to that in Caucasian population (Asian: 65.1%, 95% CI = 53.2%–77.0%; Caucasian: 30.6%, 95% CI = 25.2%–36.0%; *P = *0.003).

Overall, none of the genetic models produced significant association between rs2292832 and cancer risk. Similarly, no positive result was found in most of the subgroups, except that homozygote TT had an effect of increasing risk of other cancers compared with C allele carriers (OR = 1.388, 95% CI = 1.083–1.778, *P*_H = _0.427) and significant association with increased cancer risk was also found in small sample group for allelic contrast (OR = 1.106, 95% CI = 1.012–1.209, *P*_H = _0.461) and recessive model (OR = 1.217, 95% CI = 1.078–1.373, *P*_H = _0.380). These results are summarized in Table 5.

**Table 5:** Meta-analysis of miR-149 rs2292832 polymorphism with cancer risk.

Variables	n^a^	T vs. C				TT vs. CC				CT vs. CC				TT+CT vs. CC				TT vs. CT+CC			
		OR(95% CI)	*P*	*P-H*	*I^2^*	OR(95% CI)	*P*	*P-H*	*I^2^*	OR(95% CI)	*P*	*P-H*	*I^2^*	OR(95% CI)	*P*	*P-H*	*I^2^*	OR(95% CI)	*P*	*P-H*	*I^2^*
**Total**	12	1.022(0.966–1.082)	0.449	0.283	16.8	1.002(0.880–1.140)	0.980	0.529	0.0	0.950(0.862–1.048)	0.306	0.971	0.0	0.975(0.892–1.065)	0.571	0.979	0.0	1.082(0.990–1.183)	0.083	0.109	36.2
**Cancer type**																					
Breast Cancer	2	1.078(0.860–1.350)	0.515	0.121	58.4	1.043(0.794–1.369)	0.763	0.361	0.0	0.977(0.821–1.162)	0.789	0.907	0.0	0.991(0.839–1.169)	0.911	0.656	0.0	1.170(0.819–1.670)	0.388	0.123	58.0
Colorectal Cancer	3	1.063(0.935–1.207)	0.352	0.979	0.0	1.072(0.807–1.424)	0.631	0.619	0.0	0.856(0.660–1.109)	0.238	0.996	0.0	0.942(0.738–1.202)	0.629	0.995	0.0	1.161(0.972–1.386)	0.100	0.628	0.0
Lung Cancer	2	1.090(0.762–1.560)	0.638	0.073	68.8	1.259(0.547–2.902)	0.588	0.058	72.1	0.986(0.764–1.273)	0.915	0.562	0.0	0.995(0.782–1.266)	0.966	0.238	28.2	1.234(0.598–2.545)	0.569	0.071	69.4
SCCHN	3	0.957(0.853–1.074)	0.458	0.688	0.0	0.863(0.661–1.126)	0.277	0.619	0.0	0.976(0.826–1.154)	0.776	0.323	0.0	0.966(0.839–1.111)	0.626	0.651	0.0	0.930(0.753–1.149)	0.503	0.742	0.0
other	2	1.200(0.997–1.444)	0.054	0.259	21.6	1.181(0.790–1.767)	0.417	0.271	17.3	0.825(0.550–1.239)	0.354	0.409	0.0	0.992(0.677–1.452)	0.965	0.312	2.0	1.388(1.083–1.778)	0.010	0.427	0.0
**Ethnicity**																					
Caucasian	4	1.014(0.904–1.139)	0.810	0.220	33.9	1.261(0.749–2.123)	0.383	0.102	56.2	0.991(0.848–1.160)	0.914	0.688	0.0	1.000(0.876–1.143)	0.996	0.767	0.0	1.286(0.761–2.171)	0.348	0.082	59.9
Asian	8	1.025(0.960–1.095)	0.463	0.255	22.0	0.986(0.851–1.143)	0.852	0.745	0.0	0.925(0.817–1.048)	0.222	0.949	0.0	0.955(0.849–1.075)	0.447	0.945	0.0	1.085(0.986–1.193)	0.094	0.154	34.3
**Design**																					
HB	4	0.984(0.891–1.087)	0.756	0.599	0.0	0.901(0.718–1.130)	0.368	0.733	0.0	0.960(0.821–1.123)	0.613	0.532	0.0	0.963(0.843–1.100)	0.577	0.831	0.0	1.012(0.860–1.190)	0.890	0.451	0.0
PB	6	1.029(0.957–1.107)	0.437	0.156	37.6	1.007(0.854–1.187)	0.934	0.645	0.0	0.943(0.826–1.077)	0.387	0.923	0.0	0.970(0.855–1.101)	0.636	0.917	0.0	1.092(0.979–1.217)	0.116	0.079	49.4
**Sample size**																					
≥1000	3	0.967(0.898–1.042)	0.383	0.901	0.0	0.924(0.778–1.098)	0.370	0.892	0.0	0.984(0.877–1.104)	0.781	0.950	0.0	0.971(0.871–1.084)	0.604	0.920	0.0	0.940(0.823–1.073)	0.357	0.891	0.0
<1000	9	1.106(1.012–1.209)	0.027	0.461	0.0	1.111(0.914–1.350)	0.292	0.442	0.0	0.870(0.724–1.046)	0.138	0.957	0.0	0.981(0.845–1.139)	0.802	0.902	0.0	1.217(1.078–1.373)	0.001	0.380	0.0
**HWE**																					
Yes	10	1.029(0.970–1.091)	0.346	0.261	19.8	1.019(0.892–1.164)	0.781	0.534	0.0	0.957(0.867–1.057)	0.389	0.977	0.0	0.981(0.893–1.078)	0.693	0.973	0.0	1.095(0.997–1.202)	0.058	0.090	40.2

SCCHN: squamous cell carcinoma of the head and neck; HB: hospital based; PB: population based; HWE: Hardy-Weinberg equilibrium; OR: odds ratio; CI: confidence interval; *P*: p value; *P-H*: P value of Q for heterogeneity test; *I^2^*: 0–25%, no heterogeneity; 25–50%, modest heterogeneity; 50%, high heterogeneity;

a Number of studies involved.

Random effects model was used when P value of Q for heterogeneity test (P-H)<0.05 or *I^2^*>50%; otherwise, fixed effect model was used.

#### miR-27a rs895919

For miR-27a rs895919, we collected nine comparisons with 4662 cases and 5625 controls. No significant difference in G allele frequency between Asians and Caucasians was observed (Asian: 32,4%, 95% CI = 21.2%–43.6%; Caucasian: 32.1%, 95% CI = 28.7%–35.6%; *P = *0.949).

Overall, there was no significant association observed in all comparisons. However, in subgroup analysis, a decreased risk was found in other cancers (AG vs. AA: OR = 0.828, 95% CI = 0.698–0.982, *P*_H_ = 0.030; GG+AG vs. AA: OR = 0.821, 95% CI = 0.698–0.966, *P*_H_ = 0.017), large sample groups (G vs. A: OR = 0.875, 95% CI = 0.811–0.945, *P*_H_ = 0.001; AG vs. AA: OR = 0.806, 95% CI = 0.726–0.895, *P*_H_ <0.001; GG+AG vs. AA: OR = 0.815, 95% CI = 0.738–0.900, *P*_H_ <0.001), the Caucasian population (AG vs. AA: OR = 0.879, 95% CI = 0.792–0.975, *P*_H_ = 0.015) and population-based studies (G vs. A: OR = 0.900, 95% CI = 0.830–0.975, *P*_H_ = 0.010) (Table 6).

**Table 6:** Meta-analysis of miR-27a rs895919 polymorphism with cancer risk.

Variables	n^a^	G vs. A				GG vs. AA				AG vs. AA				GG+AG vs. AA				GG vs. AG+AA			
		OR(95% CI)	*P*	*P-H*	*I^2^*	OR(95% CI)	*P*	*P-H*	*I^2^*	OR(95% CI)	*P*	*P-H*	*I^2^*	OR(95% CI)	*P*	*P-H*	*I^2^*	OR(95% CI)	*P*	*P-H*	*I^2^*
**Total**	9	0.945(0.853–1.048)	0.284	0.008	61.1	0.897(0.714–1.127)	0.352	0.017	56.9	0.980(0.836–1.149)	0.805	0.001	68.6	0.959(0.828–1.112)	0.581	0.002	67.1	0.891(0.722–1.101)	0.286	0.023	55.0
**Cancer type**																					
Breast Cancer	4	0.930(0.864–1.002)	0.056	0.155	42.7	0.915(0.775–1.081)	0.295	0.419	0.0	1.009(0.788–1.292)	0.944	0.003	78.5	0.977(0.797–1.199)	0.825	0.014	71.6	0.929(0.793–1.087)	0.357	0.165	41.0
Gastric Cancer	2	1.029(0.587–1.803)	0.922	0.001	90.9	0.717(0.122–4.227)	0.713	<0.001	92.6	1.152(0.765–1.734)	0.499	0.078	67.8	1.116(0.629–1.977)	0.708	0.009	85.2	0.668(0.139–3.220)	0.616	0.001	91.0
other	3	0.862(0.759–0.979)	0.022	0.513	0.0	0.794(0.586–1.077)	0.139	0.643	0.0	0.828(0.698–0.982)	0.030	0.153	46.7	0.821(0.698–0.966)	0.017	0.259	26.1	0.865(0.645–1.161)	0.335	0.512	0.0
**Ethnicity**																					
Caucasian	4	0.929(0.862–1.002)	0.055	0.157	42.4	0.924(0.780–1.094)	0.359	0.421	0.0	0.879(0.792–0.975)	0.015	0.116	49.2	0.914(0.782–1.068)	0.258	0.099	52.1	0.983(0.836–1.156)	0.84	0.647	0.0
Asian	5	0.938(0.769–1.144)	0.526	0.004	74.0	0.788(0.485–1.280)	0.336	0.004	74.4	1.064(0.789–1.435)	0.685	0.001	77.8	1.008(0.762–1.334)	0.954	0.002	77.1	0.744(0.480–1.154)	0.187	0.006	72.4
**Design**																					
HB	5	0.938(0.765–1.149)	0.536	0.004	73.9	0.801(0.483–1.329)	0.390	0.003	0.003	0.964(0.760–1.223)	0.762	0.022	65.0	0.946(0.739–1.212)	0.662	0.008	70.8	0.819(0.529–1.270)	0.373	0.013	68.5
PB	3	0.900(0.830–0.975)	0.010	0.745	0.0	0.864(0.722–1.034)	0.112	0.970	0.0	0.978(0.726–1.316)	0.881	0.004	81.9	0.921(0.742–1.143)	0.455	0.039	69.2	0.889(0.750–1.053)	0.174	0.197	38.4
**Sample size**																					
≥1000	3	0.875(0.811–0.945)	0.001	0.575	0.0	0.855(0.717–1.018)	0.078	0.936	0.0	0.806(0.726–0.895)	<0.001	0.200	37.9	0.815(0.738–0.900)	<0.001	0.296	17.8	0.944(0.798–1.117)	0.503	0.790	0.0
<1000	6	1.008(0.857–1.184)	0.927	0.017	63.6	0.895(0.583–1.374)	0.611	0.004	71.0	1.127(0.985–1.291)	0.082	0.186	33.4	1.087(0.956–1.236)	0.205	0.080	49.2	0.817(0.547–1.220)	0.323	0.004	70.9
																					

HB: hospital based; PB: population based; OR: odds ratio; CI: confidence interval; *P*: p value; *P-H*: P value of Q for heterogeneity test; *I^2^*:0–25%, no heterogeneity; 25–50%, modest heterogeneity; 50%, high heterogeneity;

a Number of studies involved. Random effects model was used when P value of Q for heterogeneity test (P-H) <0.05 or *I^2^*>50%; otherwise, fixed effect model was used.

### Test of Heterogeneity

Heterogeneity between studies was observed in overall comparisons and subgroup analyses across the studies of rs2910164, rs11614913, rs3746444, and rs895919. Then we evaluated the source of heterogeneity for allelic contrast by cancer type, ethnicity, source of controls and sample size. For rs2910164, cancer type (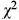 = 51.58, df = 11, *P*<0.001), ethnicity (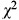 = 24.43, df = 1, *P*<0.001) and control type (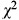 = 29.55, df = 3, *P*<0.001) provided potential sources of between-study heterogeneity. For rs11614913, cancer type (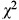 = 17.84, df = 8, *P = *0.002) and control type (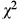 = 13.08, df = 3, *P = *0.004) was found to contribute to substantial heterogeneity. For rs3746444, ethnicity (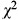 = 4.92, df = 1, *P = *0.027) and sample size (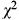 = 4.6, df = 1, *P = *0.032) contributed substantially to heterogeneity. For miR-27a rs895919, sample size (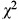 = 5.74, df = 1, *P = *0.017) was the main source of between-study heterogeneity.

### Sensitivity Analysis

Influence of each study involved in the meta-analysis on the pooled ORs for each of the studied SNPs was examined by repeating the meta-analysis and omitting each study one at a time. The corresponding pooled ORs were not materially altered.

### Publication Bias

We conducted Begg’s funnel plot and Egger’s test to assess the publication bias of included studies for all the SNPs. For miR-146a rs2910164 (Figure S1), miR-196a2 rs11614913 (Figure S2) and miR-499 rs3746444 (Figure S3), no evidence of publication bias was suggested in the results from the Begg’s funnel plot and Egger’s test for allelic contrast. Similar results were observed in other models (data not shown). However, for miR-149 rs2292832 (Figure S4), significant publication bias was found in allelic contrast (*P* = 0.006), homozygote comparison (*P* = 0.005) and the recessive model (*P* = 0.007). For miR-27a rs895919 (Figure S5), no evidence of publication bias was detected for allelic contrast, but publication bias was found in the heterozygote comparison (*P* = 0.039), probably due to the small number of included studies.

## Discussion

In the present study, we performed five independent meta-analyses to investigate the association between cancer risk and polymorphisms in miRNA (miR-146a rs2910164, miR-196a2 rs11614913, miR-499 rs3746444, miR-149 rs2292832, and miR-27a rs895919). The results demonstrated that the rs2910164 C allele or CC genotype was a protective factor for bladder cancer, prostate cancer, cervical cancer and colorectal cancer, but a risk factor for PTC and SCCHN. The significant association between rs2910164 and cancer risk was observed in both Asians and Caucasians, although in opposite directions. The miR-196a2 rs11614913 T allele was observed to be significantly associated with reduced cancer risk, especially for lung cancer and colorectal cancer, particularly in the Asian population. The miR-499 rs3746444 C allele increased cancer risk in the allelic contrast model and in the dominant model, especially in breast cancer. Nevertheless, this association was only observed in Asians, not in Caucasians. On the other hand, mir-149 rs2292832 and miR-27a rs895919 were not significantly related to cancer susceptibility.

Several meta-analyses have been conducted on a single miRNA SNP [88]–[110] or several miRNA SNPs [21], [111]–[120] associated with the risk of cancer(s). However, none of the meta-analyses have comprehensively covered all the studies on a particular miRNA SNP or all the commonly studied miRNA SNPs. In this study, we included all the papers published to date on the five commonly studied miRNA SNPs associated with cancer susceptibility, and in so doing incorporated more studies and cancer types than the previously published meta-analyses. For example, compared to the recently published meta-analysis by He *et al*. [112], our paper included several new studies for each of the miRNA SNPs. For mir-146a rs2910164, 19 new studies were added; for mir-196a rs11614913, 11 new studies were added; for mir-499 rs3746444, 6 new studies were added; and for mir-149 rs2292832, 5 new studies were added. In addition, we analyzed 9 case-control studies on miR-27a rs895919, which were not included in the meta-analysis by He *et al*. [112]. Thus, to the best of our knowledge, the present study is the most comprehensive and robust meta-analysis when compared to previously published meta-analyses in this field [21], [88]–[120].

The rs2910164 (miR-146a) locus resides at position +60 relative to the first nucleotide of the pre-miR-146a gene. This polymorphism presents as a change from G to C in the passenger strand, resulting in a change from the G:U pair to the C:U mismatch in the stem structure of the miR-146a precursor [17]. The C-allelic miR-146a precursor has lower transcriptional activity than the G-allele due to decreased nuclear primiR-146a processing efficiency; this leads to low levels of mature miR-146a and affects target mRNA binding [84], [85]. The decreased amount of miR-146a reduces the inhibition of target genes involved in the Toll-like receptor and cytokine signaling pathway (TRAF6, IRAK1) and impaired nuclear factor (NF)-κB activity [85], [121]. Studies have shown that miR-146a plays an important role in cell proliferation and metastatic ability in some cancers and that its deregulation is possibly involved in carcinogenesis [84], [85], [121]–[123]. However, the meta-analysis results suggested no significant association between this polymorphism and cancer susceptibility in the overall pooled result. In the case of subgroup analysis divided by cancer type, the rs2910164 C allele was associated with a decreased risk of bladder cancer, cervical cancer, colorectal cancer and prostate cancer, but an increased risk of PTC and SCCHN. In contrast to the previously published results by He *et al*. [112], no significant association was found between rs2910164 and HCC or ESCC. These results suggest that the association between the miR-146a rs2910164 polymorphism and cancer susceptibility was cancer-type dependent. The potential explanation for this phenomenon may be that different cancers have differing pathogenesis. In addition, we found that the association between the rs2910164 polymorphism and cancer risk was ethnicity dependent, as supported by Wang *et al.* [88]. This may be due to the difference in genetic backgrounds among races due to allele frequency or various carcinogenic mechanisms at tumor sites; another possibility may be that the polymorphism may be in linkage disequilibrium with the causal variant [124]. In contrast to our results, He *et* *al*. [112] found no association between this polymorphism and cancer risk among Caucasians.

MiR-196a2 is composed of two different mature miRNAs (miR-196a-5P and miR-196a-3P), which are processed from the same stem-loop [125]. rs11614913, located in the mature sequence of miR-196a-3P, could influence the production levels of mature miR-196a and could have an impact on the expression of its target gene. Therefore, the altered expression patterns of miR-196a could influence its potential targets, which may play a role in regulating carcinogenesis. Previous meta-analysis studies have suggested an association between rs11614913 and the risk of cancers [21], [91]–[92], [112]–[114]. The present meta-analysis also provides evidence that the miR-196a2 rs11614913 T allele is significantly associated with reduced cancer risk in the allelic contrast, the homozygote comparison, and the recessive models, similar to the findings of previous studies [112]–[114]. In the subgroup analysis that was divided by cancer type, homozygote TT had the effect of decreasing the risk of lung cancer and colorectal cancer compared with that for CC homozygote or C allele carriers. T allele carriers also showed decreased cancer susceptibility compared with homozygote CC carriers in liver cancer, whereas Wang *et al.* [111] and He *et al*. [112] reported that this polymorphism has no association with the risk of HCC. Moreover, no association was found between miR-196a2 rs11614913 and bladder cancer, breast cancer, gastric cancer, ESCC, or SCCHN. Guo *et al.* [89] and Wang *et al.* [93] found that the C allele could increase cancer risk in gastric cancer. In ethnic subgroup analysis, a strong association was found between rs11614913 and cancer risk among Asians but not among Caucasians, which was similar to the findings of previous studies [112]–[113]. In addition, biochemical studies on rs11614913 confirmed the results of our meta-analysis. It has been well established that Hox gene expression is deregulated in lung and prostate cancers [126]–[127], and members of the Hox family have been found to be significantly downregulated in cells treated with pre-miR-196a-C [127]. Two tumor suppressors (GADD45G and INHBB) were reported to be downregulated and several oncogenes (TP63 and genes encoding two calcium-binding proteins) were found to be upregulated in breast cancer cells after pre-miR-196a-C introduction, suggestive of the oncogenic activity of pre-miR-196a-C and protective role of pre-miR-196a-T [83], [128]. Our results provide compelling evidence that the miR-196a2 rs11614913 polymorphism plays a crucial role in the development of cancer. Screening patients harboring the miR-196a2 rs11614913 polymorphism may prove clinically useful for the prediction and prevention of cancer.

The miR-499T>C (rs3746444) polymorphism has been identified within the stem region of the mir-499 gene and results in an A:U to G:U mismatch in the stem structure of the miR-499 precursor. The presence of this mismatch would affect Sox6 and Rod1 genes, which are important for the etiology of cancers [72], [129]. Several studies have identified miR-499 rs3746444 as a possible biomarker for multiple cancers [20], [70], [71], [79], [82]; however, the mechanism by which this occurs remains unknown. Our results showed that the rs3746444 C allele could increase cancer risk in the allelic contrast model and in the dominant model, which was consistent with the results of Srivastava *et al.* [114]. In analysis stratified by cancer type, significant associations between the rs3746444 polymorphism and cancer risk were observed for breast cancer, which is in contrast to the results reported by Srivastava *et al*. [114] and He *et al*. [112]. However, no significant result was observed for other cancers under any genetic model. Subgroup analysis by ethnicity showed that the C allele was associated with increased cancer risk in the Asian population, but not in the Caucasian population.

For mir-149 rs2292832, a significant association was found only in some of the subgroup analyses but not in the pooled results. rs2292932 in miR-149 has been tested for several cancers but was not found to be associated with cancer risk [61], [71], [81], [82], [90], [112], [114]. This suggests that the molecular mechanisms underlying the genetic associations of miRNA-SNPs with cancer risk may be complex and variable. Our results should be interpreted with caution, considering that the influence of the T allele in miR-149 might be masked by the presence of other unidentified causal genes involved in cancer development [90] and the limited number (12) of studies on this polymorphism. More studies will need to be analyzed to confirm the results.

MiR-27a rs895919 is located in the terminal loop of pre-miRNA-27a (an intergenic region of chromosome 19), which is upregulated in many tumors [130] and has been considered to be an oncomir [131]–[133]. To date, several epidemiologic studies have been conducted to investigate the association between the rs895919 polymorphism and cancer risk [26]–[28], [73], [74]; however, the results remain inconsistent and inconclusive. The results of two previous meta-analyses have indicated that the G allele in miR-27a rs895819 may be associated with decreased risk for some cancers, as well as with reduced cancer risk in Caucasians to some extent [94], [95]. Based on our study, no association was observed between this polymorphism and cancer risk when all the data were pooled in the meta-analysis. Our results also showed that the rs895819 G allele was associated with decreased cancer risk in a Caucasian population, but was inconsistent with the abovementioned two articles on cancer type. Because of the limited number (9) of studies on this polymorphism, the results should be interpreted with caution.

Nevertheless, our study still has some limitations. First, relatively large heterogeneity was observed across some studies, which could be due to the difference in cancer types, the geographic areas (environmental factors), and genetic backgrounds of the samples. Second, the relatively small sample size of studies for some SNPs may lead to low statistical power, especially in stratified analysis. Third, lack of original data from the reviewed studies restricted further evaluation of potential interactions; this is of particular importance because gene–gene and gene–environment interactions may modulate various disease risks. Fourth, our analysis was limited to Asian and Caucasian ethnicities; therefore, it is uncertain whether these results can be generalized to other populations. Fifth, restriction to studies published in English or Chinese might confer potential language bias; moreover, publication bias might also exist because only published studies were included in this meta-analysis, and studies with no statistically significant results often have less chance for publication.

In conclusion, our results suggest that the miR-146a rs2910164 C allele is a protective factor for bladder cancer, prostate cancer, cervical cancer, and colorectal cancer in Asians, whereas it is a risk factor for PTC and SCCHN in Caucasians. mir-196a2 rs11614913 has significant association with overall cancer risk, especially for lung cancer, colorectal cancer, and other cancers in the Asian population. We also found that the mir-499 rs3746444 polymorphism could increase cancer risk in the Asian population. However, no significant association was observed between mir-149 rs2292832 and miR-27a rs895919 and overall cancer risk. Further studies with a larger sample size will be needed to clarify the possible roles of these polymorphisms in different kinds of cancers.

## Supporting Information

Figure S1 **Begg’s funnel plot of publication bias for miR-146a rs2910164 G>C: C vs. G.** Each point represents a separate study for the indicated association. Log[or], natural logarithm of OR. Horizontal line, mean effect size.
(TIF)

Figure S2 **Begg’s funnel plot of publication bias for miR-196a2 rs11614913 C>T: T vs. C.** Each point represents a separate study for the indicated association. Log[or], natural logarithm of OR. Horizontal line, mean effect size.
(TIF)

Figure S3 **Begg’s funnel plot of publication bias for miR-499 rs3746444 T>C: C vs. T.** Each point represents a separate study for the indicated association. Log[or], natural logarithm of OR. Horizontal line, mean effect size.
(TIF)

Figure S4 **Begg’s funnel plot of publication bias for miR-149 rs2292832 C>T: T vs. C.** Each point represents a separate study for the indicated association. Log[or], natural logarithm of OR. Horizontal line, mean effect size.
(TIF)

Figure S5 **Begg’s funnel plot of publication bias for miR-27a rs895919A>G: G vs. A.** Each point represents a separate study for the indicated association. Log[or], natural logarithm of OR. Horizontal line, mean effect size.
(TIF)
