# Supplementary figures and images for: Association between microRNA Polymorphisms and Cancer Risk Based on the Findings of 66 Case-Control Studies
Source: PLoS One. 2013 Nov 20;8(11):e79584. doi: 10.1371/journal.pone.0079584 (PMC3835861; doi:10.1371/journal.pone.0079584)

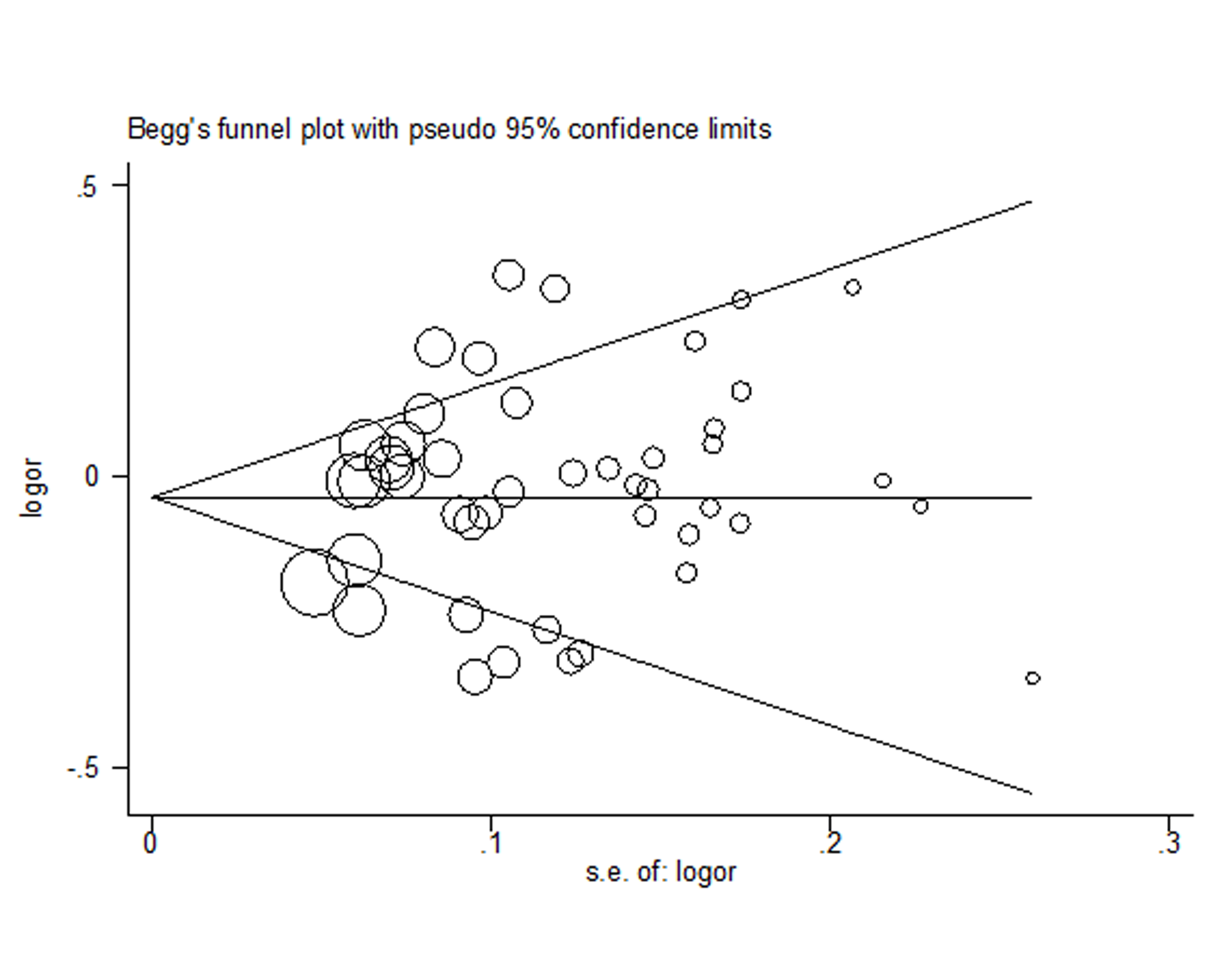

Supplement: Figure S1 — Begg’s funnel plot of publication bias for miR-146a rs2910164 G>C: C vs. G. Each point represents a separate study for the indicated association. Log[or], natural logarithm of OR. Horizontal line, mean effect size. (TIF) [file pone.0079584.s001.tif]

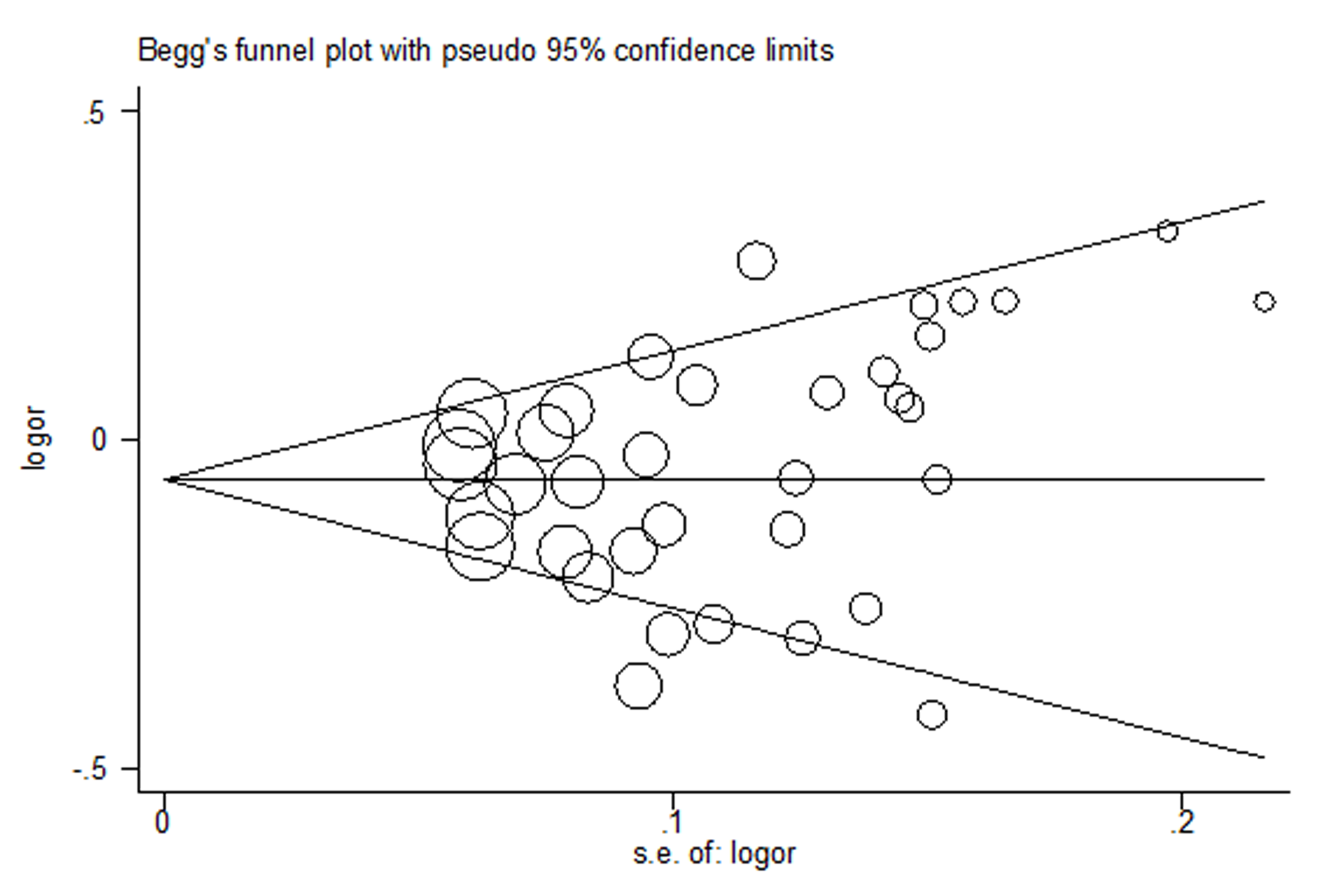

Supplement: Figure S2 — Begg’s funnel plot of publication bias for miR-196a2 rs11614913 C>T: T vs. C. Each point represents a separate study for the indicated association. Log[or], natural logarithm of OR. Horizontal line, mean effect size. (TIF) [file pone.0079584.s002.tif]

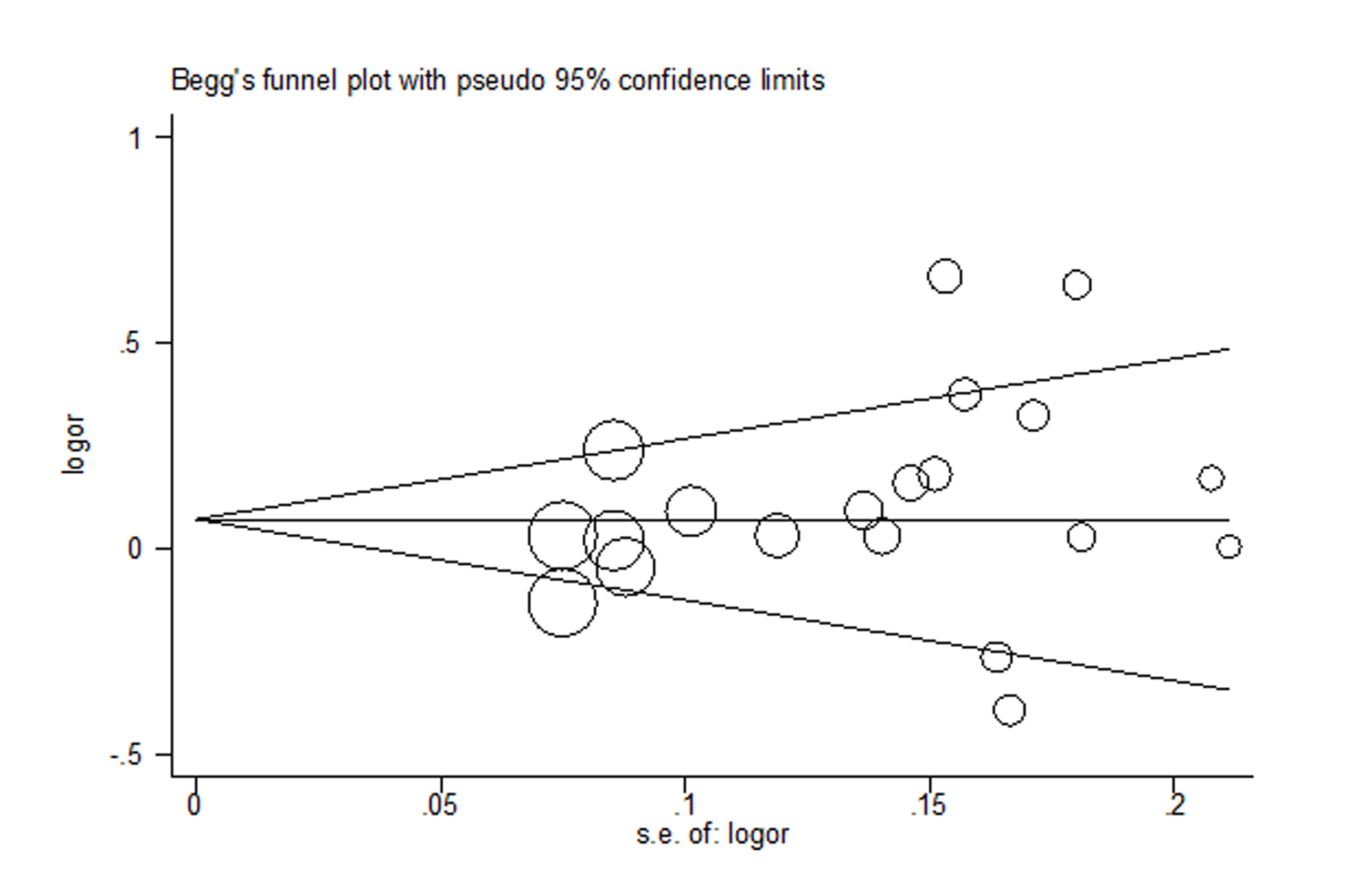

Supplement: Figure S3 — Begg’s funnel plot of publication bias for miR-499 rs3746444 T>C: C vs. T. Each point represents a separate study for the indicated association. Log[or], natural logarithm of OR. Horizontal line, mean effect size. (TIF) [file pone.0079584.s003.tif]

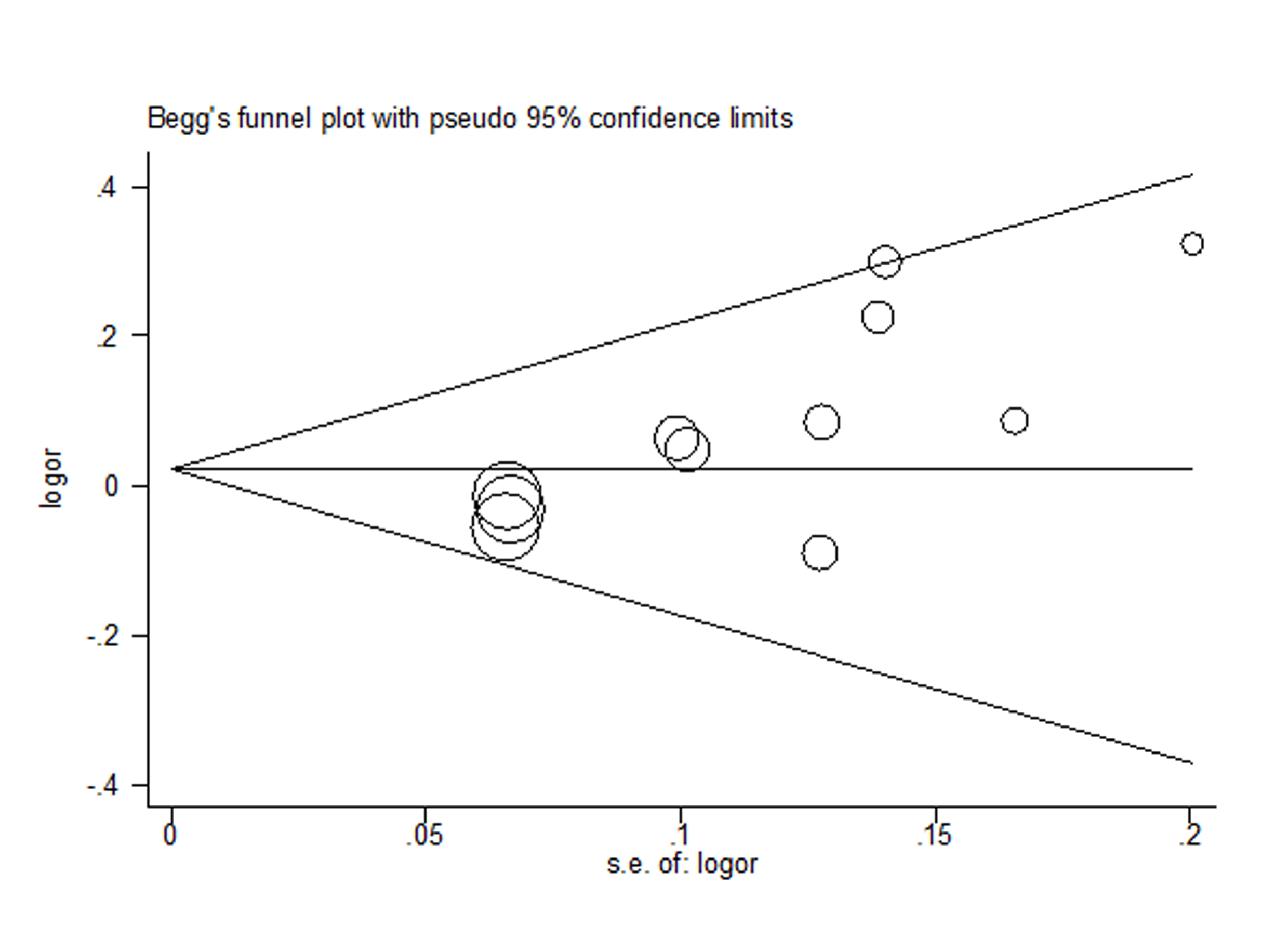

Supplement: Figure S4 — Begg’s funnel plot of publication bias for miR-149 rs2292832 C>T: T vs. C. Each point represents a separate study for the indicated association. Log[or], natural logarithm of OR. Horizontal line, mean effect size. (TIF) [file pone.0079584.s004.tif]

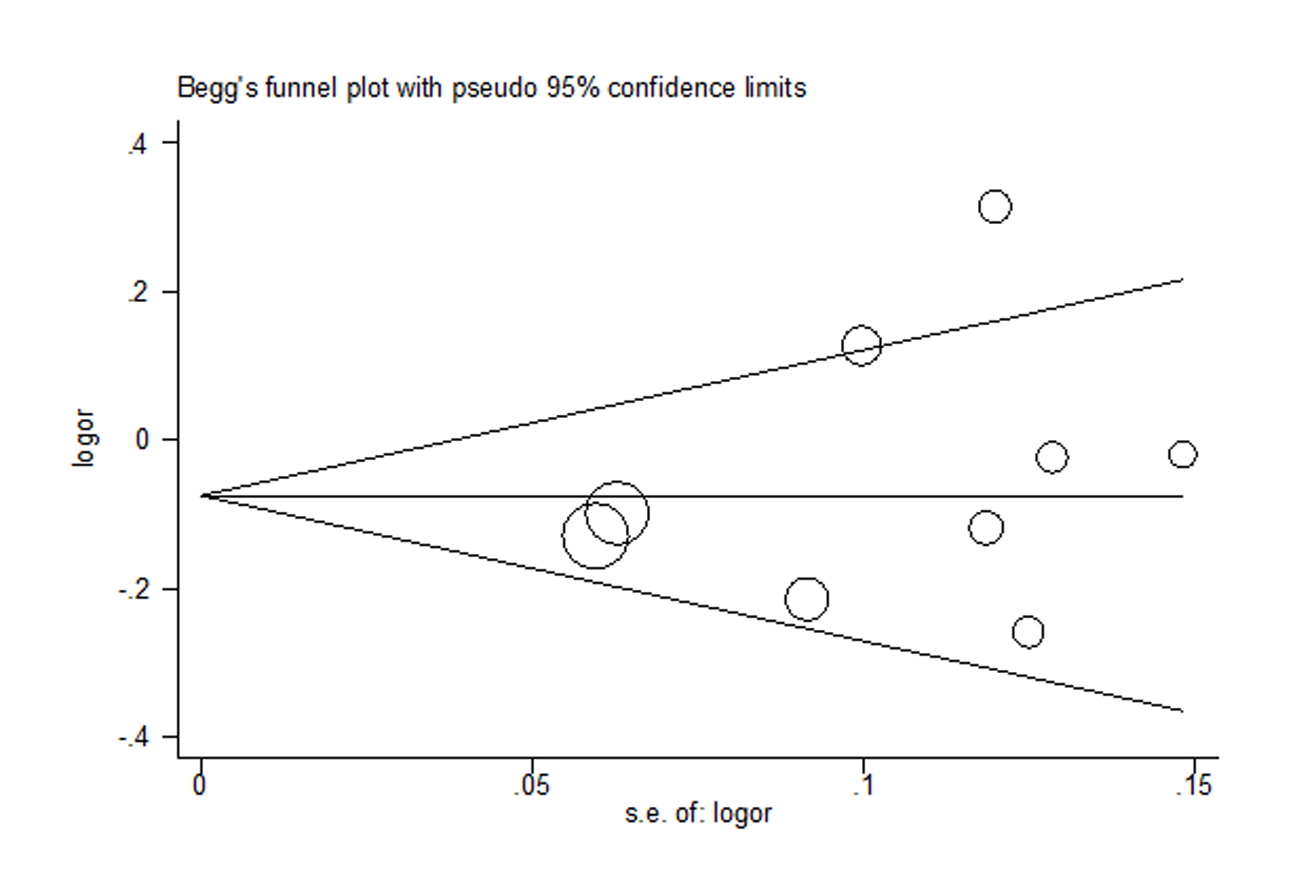

Supplement: Figure S5 — Begg’s funnel plot of publication bias for miR-27a rs895919A>G: G vs. A. Each point represents a separate study for the indicated association. Log[or], natural logarithm of OR. Horizontal line, mean effect size. (TIF) [file pone.0079584.s005.tif]
